# N-Acyl Homoserine Lactone-Mediated Quorum Sensing Regulates Species Interactions in Multispecies Biofilm Communities

**DOI:** 10.3389/fcimb.2021.646991

**Published:** 2021-03-18

**Authors:** Sujatha Subramoni, Muhammad Zulfadhly Bin Mohammad Muzaki, Sean C. M. Booth, Staffan Kjelleberg, Scott A. Rice

**Affiliations:** ^1^ Singapore Centre for Environmental Life Sciences Engineering, Nanyang Technological University, Singapore, Singapore; ^2^ School of Biological Sciences, Nanyang Technological University, Singapore, Singapore; ^3^ ithree Institute, The University of Technology Sydney, Sydney, NSW, Australia

**Keywords:** quorum sensing, species interactions, multispecies, biofilms, *Pseudomonas aeruginosa*, *Pseudomonas protegens*, *Klebsiella pneumoniae*

## Abstract

Bacterial biofilms are important medically, environmentally and industrially and there is a need to understand the processes that govern functional synergy and dynamics of species within biofilm communities. Here, we have used a model, mixed-species biofilm community comprised of *Pseudomonas aeruginosa* PAO1, *Pseudomonas protegens* Pf-5 and *Klebsiella pneumoniae* KP1. This biofilm community displays higher biomass and increased resilience to antimicrobial stress conditions such as sodium dodecyl sulfate and tobramycin, compared to monospecies biofilm populations. *P. aeruginosa* is present at low proportions in the community and yet, it plays a critical role in community function, suggesting it acts as a keystone species in this community. To determine the factors that regulate community composition, we focused on *P. aeruginosa* because of its pronounced impact on community structure and function. Specifically, we evaluated the role of the N-acyl homoserine lactone (AHL) dependent quorum sensing (QS) system of *P. aeruginosa* PAO1, which regulates group behaviors including biofilm formation and the production of effector molecules. We found that mixed species biofilms containing *P. aeruginosa* QS mutants had significantly altered proportions of *K. pneumoniae* and *P. protegens* populations compared to mixed species biofilms with the wild type *P. aeruginosa*. Similarly, inactivation of QS effector genes, e.g. *rhlA* and *pvdR*, also governed the relative species proportions. While the absence of QS did not alter the proportions of the two species in dual species biofilms of *P. aeruginosa* and *K. pneumoniae*, it resulted in significantly lower proportions of *P. aeruginosa* in dual species biofilms with *P. protegens*. These observations suggest that QS plays an important role in modulating community biofilm structure and physiology and affects interspecific interactions.

## Introduction

Bacterial biofilms are made up of surface-attached or suspended aggregates encased in self-produced extracellular polymeric substances (EPS). Biofilms in natural systems normally contain a rich mixture of species. In addition to the species level diversity, biofilms can also contain cells that are physiologically diverse due to various chemical gradients (e.g. oxygen, carbon source etc.) in different parts of the biofilm (exposed surface vs. interior) (Davey and O’Toole G, 2000). Biofilm communities have been shown to display a number of emergent properties, including enhanced stress and antimicrobial tolerance, increased biomass and reduced genetic variation relative to comparable monospecies biofilms (Flemming et al., 2016). Such effects are not observed for planktonic communities suggesting that these behaviors are biofilm specific. Biofilm formation may enable such responses due to the ability of species to optimize their spatial organization, e.g. to colocalize with collaborating species or to avoid competitors. The biofilm matrix plays a key role in these processes, enabling and stabilizing localization. The matrix further facilitates the establishment of gradients that result in for the formation of microniches that further allow for specialized growth, e.g. growth of anaerobes under otherwise oxygenic conditions (Flemming and Wingender, 2010). In this context, it is clear that microorganisms can modify their environment for optimal growth.

Interactions between individual species that contribute to the assembly and stability of a multi-species biofilm are still not well characterized. It is also clear that, just as observed in macroscale ecosystems, successionary processes are important for microbial communities. For example, in an oral biofilm, the initial colonizer tends to be *Streptococcus* sp., followed by the bridging species *Fusobacterium nucleatum* and finally by *Porphyromonas gingivalis* (Kommerein et al., 2018). Another example is the association of *Aggregatibacter actinomycetemcomitans* utilizing lactate produced by other oral bacteria such as *Streptococcus gordonii* to form biofilms of higher biomass (Brown and Whiteley, 2007). Thus, it is clear that bacteria undergo ecological processes that are similar to those described for higher organisms.

In this study, we investigated a 3-species biofilm community consisting of *Pseudomonas aeruginosa* PAO1, *Pseudomonas protegens* Pf-5 and *Klebsiella pneumoniae* KP1. These three species are reported to occur together as a community in the gut of *Bombyx mori* and in other environments along with various bacterial species (Chazal, 1995; Anand et al., 2010). Moreover, this mixed-species biofilm is highly reproducible in structure and viable cell counts under well-defined conditions, and therefore suitable for molecular studies into community dynamics (Jackson et al., 2001; Stoodley et al., 2001; Lee et al., 2014). Previously, we showed that this mixed-species community generated higher biomass and resistance to stress conditions, such as antibiotics (tobramycin), detergent sodium dodecyl sulfate (SDS) and predation (unpublished), than individual biofilms (Lee et al., 2014). Further, these effects were only observed when the community grew in a spatially organized biofilm and were not apparent for planktonic communities. Interestingly, *P. aeruginosa*, arguably one of the best studied model biofilm forming organisms, was the least abundant, between 1-10% of the entire community. When the percentage of *P. aeruginosa* was artificially increased by substituting the wild type strain with a more competitive, natural genetic variant, many of the emergent properties, e.g. stress resistance of the community, were lost. This would argue that, despite its low overall abundance, *P. aeruginosa* exerts a disproportionate effect on the rest of the community. Such species are frequently described as keystone organisms. We therefore wanted to explore the mechanisms by which this keystone organism controls community biofilm development.

One of the mechanisms used by *P. aeruginosa* PA01 to regulate biofilm formation, maturation and interspecies interactions is quorum sensing (QS). QS is a cell-cell communication system where bacteria produce and respond to signals to coordinate gene expression at the population level. There are four primary QS gene circuits reported for *P. aeruginosa* PAO1, the AHL-dependent Las and Rhl systems and the quinolone dependent PQS and IQS signaling systems (Lee et al., 2013; Lee and Zhang, 2015). The Las QS system, which produces and responds to 3-Oxododecanoyl)-L-homoserine lactone (3-oxo-C12-HSL), is the dominant system that can regulate both the Rhl and PQS systems (Gambello and Iglewski, 1991). The Rhl system produces and responds to N-butanoyl-L-homoserine lactone (C4-HSL) while the PQS system mainly produces and responds to 2-heptyl-3-hydroxy-4-quinolone (PQS) (Ochsner and Reiser, 1995; Pearson et al., 1995; Lee and Zhang, 2015). QS controls the expression of multiple effector molecules that have been shown to play important roles in surface colonization, nutrient acquisition and virulence for monospecies biofilms. Most studies of the role of QS in species interactions have focused on cross-species signaling. For example, it has been shown that signal production by *Pantoea agglomerans* or *Erwinia toletana* could rescue virulence in a signal deficient mutant of *Pseudomonas savastanoi* (Hosni et al., 2011). Similarly, it has been shown that *P. aeruginosa* and *Burkholderia cepacia* display signal cross talk in a mouse lung infection model (Riedel et al., 2001). While there are several reports of the role of QS in controlling expression of compounds with antimicrobial activity (Moons et al., 2006; Lesic et al., 2009; Abdel-Mawgoud et al., 2010), the overall role of QS as a regulator of interspecies interactions with mixed species biofilms is relatively poorly understood, especially in the context of QS regulated effectors.

Here, we report a role for *P. aeruginosa* PAO1 AHL QS in determining the composition of the three species biofilm community consisting also of *P. protegens* Pf-5 and *K. pneumoniae* KP1. We have generated isogenic, double-mutants of *P. aeruginosa* PAO1 in Las and Rhl systems and determined its effect on composition and structural properties of the 3-species biofilm.

## Materials and Methods

### Strains, Media, and Growth Conditions

Bacteria (**Table 1**) were grown either in M9 minimal medium (48  mm Na_2_HPO_4_; 22  mm KH_2_PO_4_; 9  mm NaCl; 19  mm NH_4_Cl; 2  mm MgSO_4_; 0.1  mm CaCl_2_; and 0.04% w/v glucose) supplemented with 0.2% w/v casamino acids (M9 Cas glu minimal medium), LB medium (Lysogeny Broth; 10 g  l^−1^ NaCl; 10 g  l^−1^ tryptone; 5 g  l^−1^ yeast extract) or ABTC medium (A + B salts of Clark and Maaløe supplemented with 2.5 mg thiamine liter^-1^ and 10 mM citrate) (Clark and Maaløe, 1967). Antibiotics when required were used at the following concentrations unless otherwise stated: gentamicin 60 µg ml^-1^ (Gm; MP Biomedicals, Singapore), carbenicillin 200 µg ml^−1^ (Cb; MP Biomedicals, Singapore) and chloramphenicol 10 µg ml^-1^ (Cm; MP Biomedicals, Singapore). All *P. aeruginosa*, *P. protegens* and *K. pneumoniae* cultures were grown at room temperature (25 °C) for 24 h with shaking (200 rpm) and used for flow-cell cultivation. Overnight *Escherichia coli* cultures were grown at 37 °C with shaking (200 rpm). Overnight cultures of *P. aeruginosa* were also grown at 37 °C with shaking (200 rpm) for mutant generation and conjugation was carried out at 30 °C.

**Table 1:** Bacteria and plasmids used in this study.

Species or strain	Genotypic and phenotypic characteristics	Source or reference
*P. protegens* Pf-5^b^	Wild type	ATCC BAA-477^a^
*P. aeruginosa* PAO1	Wild type	ATCC BAA-47^a^
*K. pneumoniae* KP-1	Wild type	(Lee et al., 2014)
*E. coli* S17-1 λpir	*hsdR recA pro RP4-2* (Tc::Mu; Km::Tn7; λ pir)	(Simon et al., 1983)
*P. aeruginosa lasIrhlI*	Derivative of *P. aeruginosa* PAO1 wild type in which both *lasI* and *rhlI* are deleted	This study
*P. aeruginosa lasRrhlR*	Derivative of *P. aeruginosa* PAO1 wild type in which both *lasR* and *rhlR* are deleted	This study
*P. aeruginosa* MPAO1	Wild type for the *P. aeruginosa* transposon mutants	**(** Davey and O’Toole G, 2000 **;** Flemming et al., 2016 **)**
*aprA*	Derivative of MPAO1; PW3252; *aprA*-E10::ISlacZ/hah	**(** Davey and O’Toole G, 2000 **;** Flemming et al., 2016 **)**
*aprD*	Derivative of MPAO1; PW3244; *aprD*-D11::ISlacZ/hah	**(** Davey and O’Toole G, 2000 **;** Flemming et al., 2016 **)**
*hcnB*	Derivative of MPAO1; PW4739; *hcnB*-H03::ISphoA/hah	**(** Davey and O’Toole G, 2000 **;** Flemming et al., 2016 **)**
*lasB*	Derivative of MPAO1; PW7302; *lasB*-F10::ISphoA/hah	**(** Davey and O’Toole G, 2000 **;** Flemming et al., 2016 **)**
*phzA1*	Derivative of MPAO1; PW8143; *phzA1*-B05::ISphoA/hah	**(** Davey and O’Toole G, 2000 **;** Flemming et al., 2016 **)**
*phzM*	Derivative of MPAO1; PW8141; *phzM*-A05::ISlacZ/hah	**(** Davey and O’Toole G, 2000 **;** Flemming et al., 2016 **)**
*pqsA*	Derivative of MPAO1; PW2799; *pqsA*-H04::ISlacZ/hah	**(** Davey and O’Toole G, 2000 **;** Flemming et al., 2016 **)**
*pqsE*	Derivative of MPAO1; PW2806; *pqsE*-G04::ISlacZ/hah	**(** Davey and O’Toole G, 2000 **;** Flemming et al., 2016 **)**
*pvdR*	Derivative of MPAO1; PW5016; PA2389-C08::ISphoA/hah	**(** Davey and O’Toole G, 2000 **;** Flemming et al., 2016 **)**
*rhlA*	Derivative of *P. aeruginosa* PAO1 wild type in which *rhlA* is deleted	(Rahim et al., 2001; Pamp and Tolker-Nielsen, 2007)
*sdsA1*	Derivative of MPAO1; PW2345; *sdsA1*-F05::ISlacZ/hah	**(** Davey and O’Toole G, 2000 **;** Flemming et al., 2016 **)**
**Plasmids**	**Relevant characteristics**	**Source or reference**
pFLP2	Ap^r^; modified pUCP20T vector carrying inducible Flp recombinase and *sacB* ^+^ gene	(Hoang et al., 1998)
pRK600	Cm^r^ ColE1 *oriV* RP4 *oriT*; helper plasmid in triparental matings	(Kessler et al., 1992)
pTNS1	Ap^r^; derivative of pUX-BF13 carrying the *tnsABCD* genes	(Choi et al., 2005)
pUC18T-mini-Tn*7*T-Gm-*eyfp*	Gm^r^ on mini-Tn*7*T; mobilizable; for YFP tagging of Gm^s^ bacteria	(Choi and Schweizer, 2006)
pEX18ApGW	Ap^r^, Cm^r^; *ccdB*, *sacB* ^+^; Gateway compatible suicide vector for Pseudomonas	(Choi and Schweizer, 2005)
pK18GT	Gm^r^; Broad-host-range gene replacement vector, *sacB* ^+^	(An et al., 2010)
pUCP22notI	*Pseudomonas*-*E. coli* shuttle plasmid	(West et al., 1994)
pUCP22notI+R2	pUCP22notI carrying full length *lasR* and *rhlR* with their endogenous promoters	This study

a American Type Culture Collection (ATCC) number.

b Pseudomonas fluorescens has recently been renamed as Pseudomonas protegens (Ramette et al., 2011; Lim et al., 2013).

### Generation of *Pseudomonas aeruginosa* Quorum Sensing Mutants

To generate *P. aeruginosa* PAO1 mutants, *lasR*, *rhlI*, and *rhlR* were amplified to include positions 500 bp and 1000 bp upstream and downstream, respectively, using Phusion polymerase (New England Biolabs, NEB, Ipswich, MA, USA) and cloned into the suicide vector pK18GT (**Table 1**). For *lasI*, 500 bp flanking the 5’ and 3’ ends of the gene were amplified in the same manner and cloned into the suicide vector pEX18ApGW (primers are indicated in **Table S1**). The PCR temperature cycling conditions for amplifying the upstream and downstream regions were as follows: initial denaturation for 30 s at 98°C, followed by 35 standard cycles: denaturation at 98°C for 10 s, primer annealing for 30 s at 69°C, and primer extension at 72°C for 15 s for 500 bp or 30 s for 1000 bp. Both cloning reactions were carried out using the Gibson Assembly Cloning kit (NEB, Beverly, MA, USA). The suicide plasmid constructs were verified by sequencing using vector specific primers pK18F and pK18R (**Table S1**) and mobilized into wild type *P. aeruginosa* PAO1 either by biparental or triparental mating using pRK600 as the helper plasmid. For biparental mating, the deletion constructs in the suicide plasmids were maintained in *Escherichia coli* S17-1 λpir. The single recombinants were selected on ABTC medium containing gentamicin (60 µg ml^-1^). Mutant colonies were selected on ABTC plates containing 15% sucrose and further checked for sensitivity to gentamicin, which would be indicative of double recombination. Finally, each putative mutant was tested by PCR to confirm the in-frame deletion by PCR amplification using primers specific to flanking region (**Table S1**) and Sanger sequencing (AITbiotech, Singapore). The *lasIrhlI* mutant was generated by deleting *lasI* in the *rhlI* mutant background while the *lasRrhlR* double mutant was generated by deleting *lasR* in the *rhlR* mutant background using the same strategy.

### Fluorescent Marking of Wild-Type *Pseudomonas aeruginosa*, Quorum Sensing Mutants, and Quorum Sensing Target Mutants With *eyfp*

The *eyfp* gene was introduced as described previously (Choi and Schweizer, 2006). Briefly, overnight cultures were washed with 300 mM sucrose at room temperature to generate electrocompetent cells. These were simultaneously electroporated with 500 ng of pUC18T-mini-Tn*7*T-Gm-*eyfp* and pTNS1 and plated on LB agar containing gentamicin 100 µg ml^-1^. Positive colonies were verified by checking for the expression of *eyfp* by confocal laser scanning microscopy (CLSM) (LSM780, Carl Zeiss, Singapore) (excitation wavelength 514 nm and emission wavelength 527 nm).

### Generation of Fluorescent Protein-Tagged Gentamicin-Sensitive Strains

It was necessary to excise the gentamicin marker to enable complementation with a gentamicin resistant vector. Therefore, the gentamicin cassette was removed from the eYFP tagged wild type and QS mutants *lasRrhlR* and *lasIrhlI* as previously described (Choi and Schweizer, 2006). Briefly, electrocompetent cells were prepared by washing with 300 mM sucrose solution and electroporated with 20-50 ng pFLP2 plasmid (Choi and Schweizer, 2006). The transformants were diluted 1000 fold and plated on LB+ Cb200 plates. Single colonies were checked for antibiotic susceptibility by patching on both LB+ Gm100 and LB+ Cb200 plates. Gentamicin sensitive colonies were checked for the presence of eYFP by CLSM (LSM780, Carl Zeiss, Singapore).

### Generation of Complementation Constructs

The full length *lasR* (1220 bp) and *rhlR* (1226 bp) genes, along with their endogenous promoter regions, were amplified from the *P. aeruginosa* wild type (**Table 1**). The PCR temperature cycling conditions for amplifying the full length genes were as follows: initial denaturation for 30 s at 98°C, followed by 35 standard cycles: denaturation at 98°C for 10 s, primer annealing for 30 s at 69°C, and primer extension at 72°C for 45 s. Both *lasR* and *rhlR* fragments were ligated together by overlap splice PCR to obtain a single PCR fragment of 2446 bp. This was done by setting up a PCR reaction containing both full length gene PCR products without any primer for 10 cycles (for overlap extension) followed by the standard PCR with primers RhlRHinF2 and LasREcoR2 for the next 30 cycles (**Table S1**). This was then cloned into pUCP22notI at the *smaI* site to generate pUCP22notI+R2. The presence of both genes was confirmed by sequencing using vector specific universal primers M13F and M13R (AITbiotech, Singapore). The gentamicin sensitive *P. aeruginosa lasRrhlR* mutant was then transformed with the pUCP22notI+R2 by electroporation (25 μF, 200 Ω and 2.5 kV cm^−1^) using a Gene Pulsar (Biorad, Hercules, CA, USA).

### Phenotypes Assessed for Complementation

Swarming plates containing 0.5% w/v Bacto agar, 0.5% w/v peptone, 0.2% w/v yeast extract and 1% w/v glucose were prepared as previously described (Chang et al., 2014). Bacteria were diluted to 0.1 OD_600_ and 2 µl was inoculated at the center of the swarming agar plate. Plates were incubated for 16 h and the diameter of swarming zone was measured. Elastase activity was determined as previously described (Yin et al., 2015). Briefly, 100 µl of culture supernatant was added to 900 µl of Elastin-Congo Red (ECR) buffer (100 mM Tris, 1 mm CaCl_2_, pH 7.5) containing 20 mg ECR. This was then incubated overnight at 37˚C with shaking. The samples were then centrifuged (4000 g for 10 min at 4˚C) to remove insoluble ECR. The absorbance (OD_495_) of the supernatant was determined and sterile medium was included as a negative control. The change in absorbance was calculated to determine the change in elastase activity. Protease activity was determined by inoculating strains onto LB agar plates containing 1% skimmed milk. After incubation at 37˚C overnight, the diameter of clearance zones around the colonies, corresponding the protease activity was measured.

### Continuous Flow-Cell Cultivation of Biofilms, Imaging, and Analyses

Flow-cell biofilms were established as previously described (Lee et al., 2014). Briefly, biofilms were cultivated in 3 channel flow cells having channel dimensions 40 mm x 4 mm x 1 mm. The flow cells were supplied with M9 cas glucose at the rate of 9 ml h^-1^ per channel. Each channel was injected with bacterial suspensions adjusted to approximately 1x10^8^ cells ml^-1^. Mixed species biofilms were established by inoculating mixed cultures of *P. aeruginosa* PAO1, *P. protegens* Pf-5 and *K. pneumoniae* in the ratio of 5:5:1, respectively. Biofilms were imaged by CLSM (LSM780, Carl Zeiss, Singapore) as described (Lee et al., 2014). For each flow cell channel, 5 image stacks were acquired from the center of the channel at a distance of 5-10 mm from inlet to give a total of 15 image stacks per strain/genotype of mixed species biofilm. Three independent, replicate flow cells evaluated for each strain/combination. The image stacks were quantified using IMARIS (Bitplane AG, Belfast, UK). Statistical analyses were carried out by GraphPad Prism version 9 for windows, GraphPd software (La Jolla, California, USA, www.graphpad.com). Biofilm structure analysis was carried out with the COMSTAT 2.1 image analysis software package with default setting (Heydorn et al., 2000a; Heydorn et al., 2000b).

### Growth and Batch Biofilm Experiments

The growth of strains in M9 medium supplemented with casamino acids and glucose was monitored by inoculating in 96 well plates at an initial OD_600_ of 0.01 and incubating at room temperature for 24 h. Batch biofilm experiments were carried out as described previously (Schleheck et al., 2009). Briefly, overnight cultures of *P. aeruginosa* mutants or the wild type grown in M9 supplemented with casamino acids and glucose were adjusted to an OD_600_ of 0.01 and 1 ml of this suspension was distributed into wells of a 24 well plate. Biofilm attached to the wells of the plate was quantified by Crystal Violet (CV) assay. Briefly, the wells were washed once with 1.5 ml M9 medium to remove loosely attached cells. Staining was carried out with 1.75 ml CV solution (0.1% w/v). Unbound CV was removed by 3 washes with 2 ml M9 medium. Finally, 2 ml of absolute ethanol was used to extract bound CV by incubating on a rotary shaker 100 rpm for 10 min at RT after which time, the OD_570_ was determined for 200 µl of the ethanol extract using Tecan M200 Infinite microplate reader (Tecan Group Ltd., Männedorf, Switzerland).

### Fitness Assays for Planktonic Cultures

*In vitro* competition experiments between *P. aeruginosa* strains and *P. protegens* were carried out in M9 cas glucose medium. Overnight cultures of *P. aeruginosa* QS regulated mutants and *P. protegens* Pf-5 were diluted 100 fold and 30 fold, respectively. The different dilutions were used to ensure similar CFUs (1X10^6^ cfu ml^-1^) were present at the start of the experiment, based on comparisons of CFUs and OD (data not shown). Equal volumes of each of the *P. aeruginosa* mutants and *P. protegens* were mixed and incubated with shaking at 200 rpm at RT. Bacteria were enumerated at 0 and 24 h by dilution drop plating in duplicate. One set of plates at 37˚C allowed growth of *P. aeruginosa* strains, whereas incubation of duplicate plates at RT allowed growth of *P. protegens*. Bacteria were differentiated based on colony morphology. The fitness of each species was calculated as follows: Log[Fold change of mutant/fold change of wild type].

### Growth Competition Assays on Agar Plates

Cultures of *P. aeruginosa* strains grown at room temperature for 24 h were adjusted to OD_600_ = 0.1. A 500 μl aliquot of these bacterial suspensions were plated on LB agar plates. *P. aeruginosa* bacterial lawns were incubated for 2 h at room temperature. Cultures of *P. protegens* Pf-5 or *K. pneumoniae* KP-1 grown at room temperature for 24 h were adjusted to OD_600_ = 2 and 2 μl of each of these suspensions were spotted on the *P. aeruginosa* lawns. Plates were incubated for 2 to 3 d at room temperature. Growth inhibition zones were observed after 36-48 h. *P. aeruginosa* strains were screened for differences in the diameter of the growth inhibition zone surrounding the *P. protegens* Pf-5 or *K. pneumoniae* KP-1 colony in the middle on LB agar.

## Results

### *Pseudomonas aeruginosa* Quorum Sensing Modulates the Species Composition of Biofilm Community

**Figure 1:**
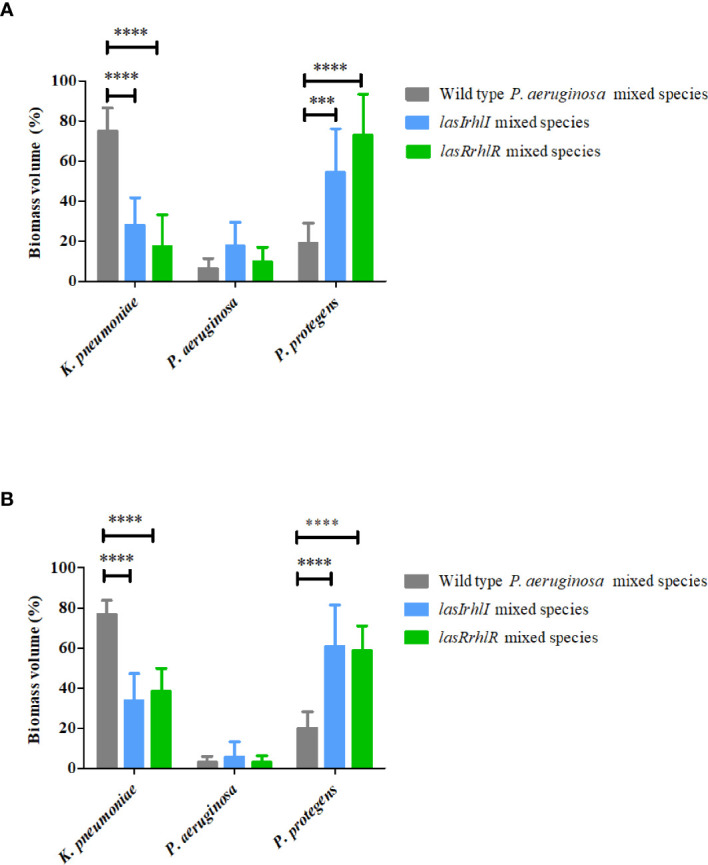
Proportions of the mixed species biofilm. The proportions of the three species in the mixed species biofilm containing either the *P. aeruginosa* wild type or the QS mutants (*lasIrhlI* or *lasrRrhlR*) after **(A)** 3 days and **(B)** 5 days of cultivation. Bar charts depict the percentages of *K*. *pneumoniae*, *P. aeruginosa*, and *P. protegens* in the wild type or QS mutant mixed species biofilm. The proportions were derived by cultivating biofilms under flow cell conditions followed by quantitative image analysis of two independent biological replicates. Error bars represent standard deviations (n = 2). *** and **** denote significant difference by two-way ANOVA followed by Tukey’s post-test (*P* < 0.001 and *P* < 0.0001).

To determine the role of *P. aeruginosa* AHL quorum sensing (QS) in the 3 species biofilm community, a comparison of wild type *P. aeruginosa* relative to the QS mutants when present as part of the mixed species biofilms was carried out after 3 and 5 d (**Figure 1**). At day 3, the biovolumes per unit area of the mutant mixed-species biofilms (16.0 ± 3.0 µm^3^ µm^-2^ for *lasIrhlI* mixed-species and 16 ± 2.6 µm^3^ µm^-2^ for *lasRrhlR* mixed-species) were similar to when the wild type *P. aeruginosa* was present in the community. This trend was similar on day 5 and the wild type mixed species biofilm had biovolumes that were similar to the QS mutants (data not shown). While the overall biovolumes were similar, the proportions of *K. pneumoniae* and *P. protegens* were significantly different for the QS mutant mixed-species biofilms compared to when the wild type *P. aeruginosa* was present in the biofilm community (**Figures 1A, B**). Quantitative image analysis of the wild type mixed species revealed that it was composed of 74.8 ± 11.7% and 76.7 ± 6.9% *K. pneumoniae*, 6.3 ± 5% and 3.1 ± 2.8% *P. aeruginosa* and 18.8 ± 10.1% and 20.1 ± 7.9% *P. protegens* at days 3 and 5, respectively. In contrast, when the signal or receptor mutants of *P. aeruginosa* were present, the relative proportions of *K. pneumoniae* decreased, with a concomitant increase in *P. protegens* and a slight increase in *P. aeruginosa* for both time points. The community with the *lasIrhlI* mutant had lower proportions of *K. pneumoniae* and higher proportions *P. protegens* compared to the wild type community (**Figures 1A, B**). After 3 d, biofilms formed with the *lasIrhlI* mutant had 28 ± 13.5% *K. pneumoniae*, 17.6 ± 11.5% *P. aeruginosa* and 54.2 ± 21.8% *P. protegens* and at 5 d, these were 33.7 ± 13.5% *K. pneumoniae*, 5.5 ± 7.6% *P. aeruginosa* and 60.7 ± 20.7% *P. protegens*. The community with the *lasRrhlR* mutant also had similar changes in proportions of *K. pneumoniae* and *P. protegens* (**Figures 1A, B**). After 3 d, biofilms formed with the *lasRrhlR* mutant had 17.3 ± 15.7% *K. pneumoniae*, 9.7 ± 7.2% *P. aeruginosa* and 72.8 ± 20.5% *P. protegens* and at 5 d, these were 38.1 ± 11.6% *K. pneumoniae*, 2.9 ± 3.3% *P. aeruginosa* and 58.8 ± 12.2% *P. protegens*. These results show that the proportions of *K. pneumoniae* and *P. protegens* were affected by a functional QS system of *P. aeruginosa*, while there was no significant change in the relative abundance of *P. aeruginosa*.

Analyses of the images of wild type and QS mutant mixed species biofilms was also performed for five parameters, microcolony size, thickness, roughness coefficient, surface to biovolume ratio and substratum coverage (**Table 2**). At both 3 and 5 d of biofilm growth, the *P. aeruginosa* wild type mixed species community had a larger microcolony size for *K. pneumoniae* when compared to *K. pneumoniae* in the *lasIrhlI* and *lasRrhlR* mixed species biofilms. Similarly, the *P. aeruginosa* wild type mixed species had thicker *K. pneumoniae* and thinner *P. protegens* biofilm structures when compared to *K. pneumoniae* and *P. protegens* in the *lasIrhlI* and *lasRrhlR* mixed species biofilms. *P. aeruginosa* thickness was not significantly different between these biofilms. The roughness coefficient provides a measure of how much the thickness of the biofilm varies. Both at 3 and 5 days of biofilm growth, the *P. aeruginosa* wild type mixed species community had a less heterogeneous *K. pneumoniae* and more heterogeneous *P. protegens* biofilm structure when compared to *K. pneumoniae* and *P. protegens* in the *lasIrhlI* and *lasRrhlR* mixed species biofilms. The surface to biovolume ratio values supports the thickness measurements. Finally, both at 3 and 5 d of biofilm growth, the *P. aeruginosa* wild type mixed species biofilms had higher substratum coverage for *K. pneumoniae* and lower substratum coverage for *P. protegens* biofilm when compared to *K. pneumoniae* and *P. protegens* in the *lasIrhlI* and *lasRrhlR* mixed species biofilms.

**Table 2:** Structural features of wild type *P. aeruginosa* and QS mutant mixed species biofilms.

Day	Channel	*P. aeruginosa* mixed species	*lasIrhlI* mixed species	*lasRrhlR* mixed species
Average microcolony size at the substratum (µm^2)				
3	*K. pneumoniae*	1055.63 ± 50.17	379.39 ± 126.26	512.55 ± 23.07
	*P. aeruginosa*	0	113.56 ± 160.61	135.20 ± 0.09
	*P. protegens*	0	1179 ± 1667.74	2,442.97 ± 733.96
5	*K. pneumoniae*	7,256.26 ± 1,584.76	2,081.06 ± 2,294.75	2,414.72 ± 2,836.66
	*P. aeruginosa*	274.27 ± 387.87	688.30 ± 973.41	222.59 ± 314.80
	*P. protegens*	385.74 ± 545.52	1,115.15 ± 501.54	902.27 ± 139.75
Average thickness (Biomass) (µm)				
3	*K. pneumoniae*	43.44 ± 1.41	39.22 ± 0.60	36.84 ± 2.12
	*P. aeruginosa*	10.29 ± 7.40	19.45 ± 2.37	13.53 ± 7.46
	*P. protegens*	12.93 ± 10.02	40.88 ± 11.48	44.78 ± 12.29
5	*K. pneumoniae*	59.54 ± 8.83	43.61 ± 1.31	42.77 ± 3.76
	*P. aeruginosa*	11.56 ± 9.07	10.75 ± 2.83	8.98 ± 4.81
	*P. protegens*	23.66 ± 3.84	37.33 ± 21.26	33.66 ± 18.62
Roughness Coefficient (Dimensionless)				
3	*K. pneumoniae*	0.5 ± 0.15	0.55 ± 0.20	0.82 ± 0.02
	*P. aeruginosa*	1.77 ± 0.12	1.74 ± 0.24	1.77 ± 0.09
	*P. protegens*	1.92 ± 0.10	0.81 ± 0.73	0.76 ± 0.34
5	*K. pneumoniae*	0.26 ± 0.10	0.47 ± 0.22	0.48 ± 0.21
	*P. aeruginosa*	1.87 ± 0.18	1.94 ± 0.07	1.84 ± 0.02
	*P. protegens*	1.73 ± 0.37	0.83 ± 0.89	0.83 ± 0.85
Surface to biovolume ratio (µm^2/µm^3)				
3	*K. pneumoniae*	1.48 ± 0.54	1.80 ± 0.06	1.92 ± 0.37
	*P. aeruginosa*	4.38 ± 0.75	3.81 ± 0.15	3.77 ± 0.34
	*P. protegens*	4.31 ± 0.78	2.15 ± 1.19	1.87 ± 0.78
5	*K. pneumoniae*	0.69 ± 0.20	1.33 ± 0.60	1.36 ± 0.47
	*P. aeruginosa*	4.35 ± 0.78	4.36 ± 0.32	4.49 ± 0.57
	*P. protegens*	4.10 ± 1.07	2.48 ± 1.56	2.60 ± 1.43
Substratum coverage (%)				
3	*K. pneumoniae*	19.51 ± 7.58	15.52 ± 4.56	15.71 ± 7.26
	*P. aeruginosa*	6.61 ± 8.65	7.77 ± 9.19	6.38 ± 6.81
	*P. protegens*	5 ± 4.72	16.48 ± 17.02	20.48 ± 11.04
5	*K. pneumoniae*	40.01 ± 8.23	26.59 ± 7.93	26.78 ± 3.39
	*P. aeruginosa*	7.54 ± 8.36	6.05 ± 6.36	6.30 ± 7.01
	*P. protegens*	5.55 ± 4.42	14.36 ± 8.69	14.85 ± 9.59

### *Pseudomonas aeruginosa* Quorum Sensing Modulates Interaction With *Pseudomonas protegens* and Not *Klebsiella pneumoniae* in Dual-Species Biofilms

**Figure 2:**
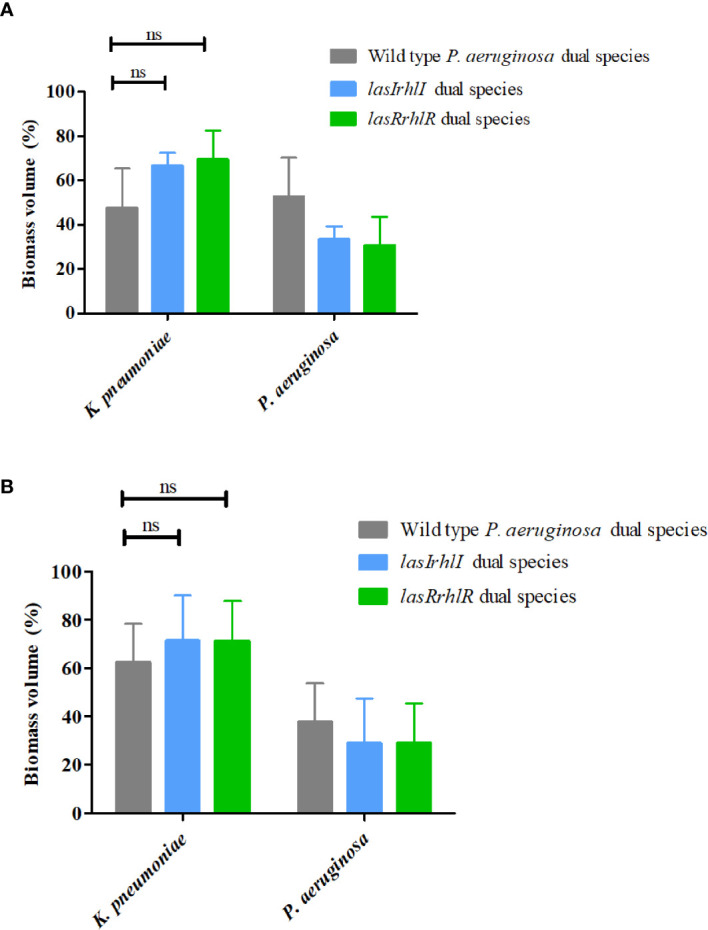
Proportions of *K. pneumoniae*-*P. aeruginosa* dual-species biofilm. The proportions of the two species in the dual species biofilm containing either the *P. aeruginosa* wild type or the QS mutants (*lasIrhlI* or *lasrRrhlR*) after **(A)** 3 days and **(B)** 5 days of cultivation. Bar charts depict the percentages of *K. pneumoniae* and *P. aeruginosa* in the wild type or QS mutant containing dual species biofilms. The proportions were derived by cultivating biofilms under flow cell conditions followed by quantitative image analysis of two independent biological replicates. Error bars represent standard deviations (n = 2). ns denotes there were no significant differences.

We hypothesized that the effect of *P. aeruginosa* QS on community composition could be due to specific interactions with the other two species in the community. Therefore, dual-species biofilms of *P. aeruginosa* wild type with *P. protegens* and *P. aeruginosa* wild type with *K. pneumoniae* were cultivated separately and compared to biofilms where the wild type *P. aeruginosa* was substituted with the isogenic QS mutants (**Figure 2**). After 3 d, the proportions were 52.5 ± 17.8% wild type *P. aeruginosa* and 47.4 ± 17.8% *K. pneumoniae*. When either of the QS mutants were introduced, the proportions of *K. pneumoniae* increased slightly, but not significantly, with a similar reduction in the proportion of the mutants. When cultivated for 5 d, we again saw only a slight increase in *K. pneumoniae* when cultivated with the QS mutants compared to when the wild type was present. This shows that QS does not drive the interactions between *P. aeruginosa* and *K. pneumoniae*, as defined by their relative proportions, between these two species under these conditions (**Figures 2A, B**).

**Figure 3:**
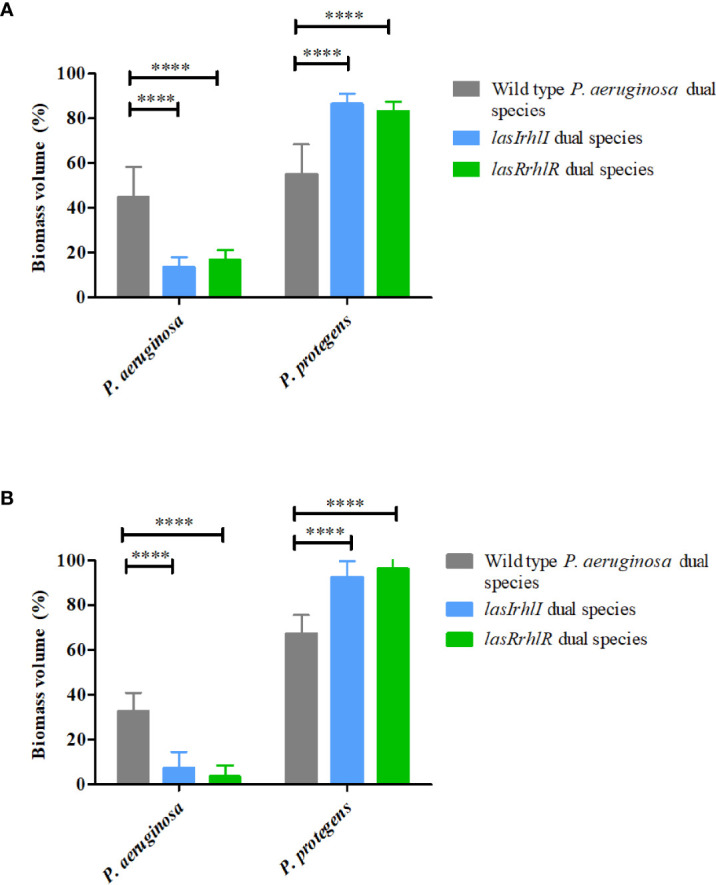
Proportions of *P. protegens*–*P. aeruginosa* dual-species biofilm. The proportions of the two species in the dual species biofilm containing either the *P. aeruginosa* wild type or the QS mutants (*lasIrhlI* or *lasrRrhlR*) after **(A)** 3 days and **(B)** 4 days of cultivation. Bar charts depict the percentages of *P. protegens* and *P. aeruginosa* in the wild type or QS mutant dual species biofilm. The proportions were derived by cultivating biofilms under flow cell conditions followed by quantitative image analysis of two independent biological replicates. Error bars represent standard deviations (n =2). **** denote significant difference by two-way ANOVA followed by Tukey’s post-test (*P* < 0.0001).

In contrast, deletion of QS in *P. aeruginosa* led to significant changes in the composition of dual species biofilms in the presence of *P. protegens*. When the wild type *P. aeruginosa* was present, it accounted for approximately 40% of the biofilm biomass with the remaining ~60% contributed by *P. protegens* at 3 and 4 d (**Figures 3A, B**). In contrast, on day 3 the proportions of the *lasRrhlR* and *lasIrhlI* mutants in the dual-species biofilms were significantly lower (13.5 ± 4.4% and 16.9 ± 4.3%, respectively), with the remaining 83-85% contributed by *P. protegens*. By day 4, the QS mutants were present at only 4-8%. Collectively, these results show that *P. aeruginosa* and *P. protegens* exhibit a competitive interaction that is controlled by the *P. aeruginosa* QS system.

Complementation of the QS mutants showed full restoration of phenotypes such as swarming, elastase activity and protease activity to wild type levels (**Figures S1A–C**). Dual species biofilms were also formed with *lasRrhlR* mutant carrying the complementation vector, pUC22notI+R2, for *P. aeruginosa*-*P. protegens* dual species biofilms (**Figure S2**). After 3 d, the dual species biofilm containing the complemented *lasRrhlR* mutant had biovolumes of 24.4 ± 17.9% *lasRrhlR*/pUCP22notI+R2 and 75.5 ± 17.3% *P. protegens*, respectively. Therefore, the complemented *lasRrhlR* mutant formed dual species biofilms that were not significantly different from, but slightly lower than, when the wild type was present with *P. protegens*.

### Quorum Sensing-Regulated Effectors Play a Cumulative Role in Interspecies Interactions

QS regulated genes in *P. aeruginosa* include those coding for secondary metabolites (phenazines, PQS), virulence factors, nutrient acquisition (elastase, protease, pyoverdine) and toxins (HCN, exotoxin) among others. It is possible that one or more of these provide fitness advantage to *P. aeruginosa* wild type in the presence of *P. protegens*. We therefore tested a range of mutants for their impact on monospecies biofilm formation and growth. These mutants were chosen as they represent some of the well-characterized genes of the Las, Rhl and Pqs regulon. Most of the mutants tested, including *aprA*, *hcnB*, *lasB, phzA1, phzM, pqsA, pqsE, pvdR*, *rhlA* and *sdsA1*, showed no significant difference in growth or monospecies biofilm formation (**Figures S3** and **S4**). Only *aprD* showed a significant reduction in monospecies biofilm formation. There was also no difference in the fitness of these mutants relative to the wild type *P. aeruginosa* when grown in the presence of *P. protegens* during planktonic growth. (**Figure S5**).

**Figure 4:**
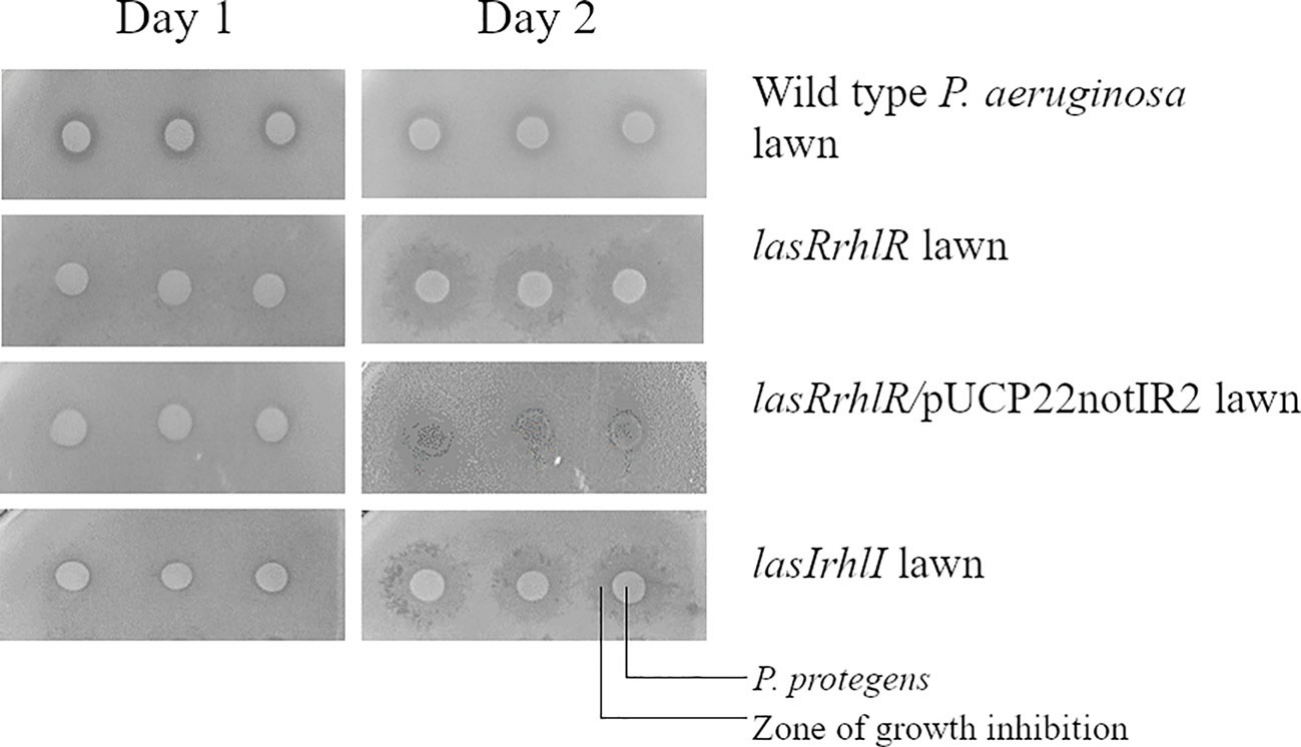
*Pseudomonas aeruginosa* mutants affected in competition with *P. protegens*. *P. protegens* was spotted on a lawn of *P. aeruginosa* wild type, QS mutants or complemented strains on LB agar plate. This panel shows the plate phenotype on day 1 and day 2 and the inhibition zone is observed from the second day.

In order to pinpoint which QS effector was involved in competitive interaction of *P. aeruginosa* with *P. protegens* and to screen a larger number of mutants, we carried out a growth inhibition assay on agar plates. Based on this screening, the *lasIrhlI* and *lasRrhlR* mutants showed an altered inhibition zone in the presence of *P. protegens* when compared to *P. aeruginosa* wild type. This sensitivity of the *lasRrhlR* mutant was abolished by providing the wild type copies of both genes in *trans* in the complemented strain (**Figure 4**). No such zone of inhibition was found when these mutants were tested in the presence of *K. pneumoniae* (**Figure S6**). Furthermore, our results also revealed that none of the mutants of QS effectors tested showed this altered inhibition zone in the presence of *P. protegens* (**Figure S7**).

**Figure 5:**
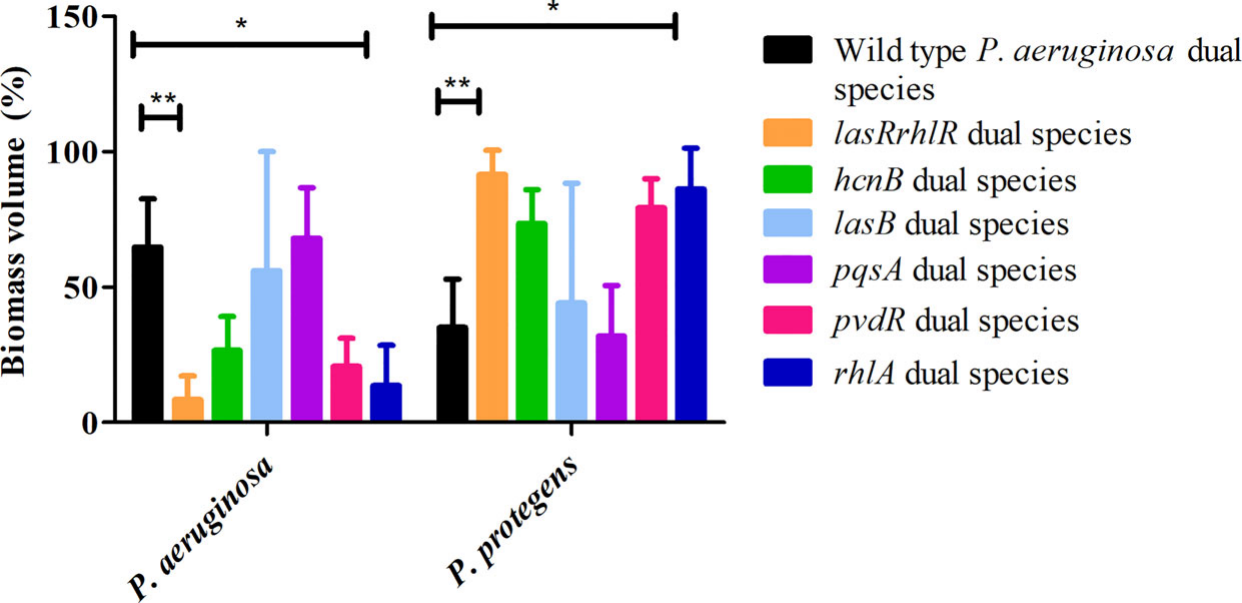
Proportions of *P. protegens* -* P. aeruginosa* dual species biofilm containing mutants of QS regulated effectors. The proportions of the two species in the mixed species biofilm containing either the *P. aeruginosa* wild type, the QS mutant *lasRrhlR* or the QS target mutants *hcnB*, *lasB*, *pvdR*, *rhlA*, or *pqsA* after 3 days of cultivation. Bar charts depict the percentages of *P. aeruginosa* and *P. protegens* in the wild type or the mutant dual species biofilms. The proportions were derived by cultivating biofilms under flow cell conditions followed by quantitative image analysis of two independent biological replicates. Error bars represent standard deviations (n = 2). * and ** denote significant difference by two-way ANOVA followed by Tukey’s post-test (*P* < 0.05; *P* < 0.01).

Since flow-cell conditions used for cultivation of biofilms are very different from agar plates, we decided to evaluate the roles of a subset of these QS effectors in dual and 3-species biofilms. First, we tested mutants of various effector genes belonging to the Las, Rhl and Pqs systems in dual species biofilms with *P. protegens*. For the *P. aeruginosa* wild type and *lasRrhlR* communities the proportions of *P. aeruginosa* were 64.8 ± 17.7% and 8.4 ± 8.8%, respectively, whereas the proportions of *P. protegens* were 35.1 ± 17.3% and 91.5 ± 8.8%, respectively as expected. Although, the proportions of *P. aeruginosa*, 20.6 ± 10.5% and 13.6 ± 14.8%, respectively, were lower for the communities containing mutants of *pvdR* and *rhlA* compared to dual species biofilms with *P. aeruginosa* wild type, only *rhlA* was significantly different (**Figure 5**). The proportions of *P. protegens* was also much higher (79.3 ± 10.5% and 86.3 ± 14.8%) for these two mutant communities compared to wild type community. The proportions of *P. aeruginosa* and *P. protegens* in *hcnB*, *lasB* and *pqsA* communities were not significantly different when compared to the wild type community.

**Figure 6:**
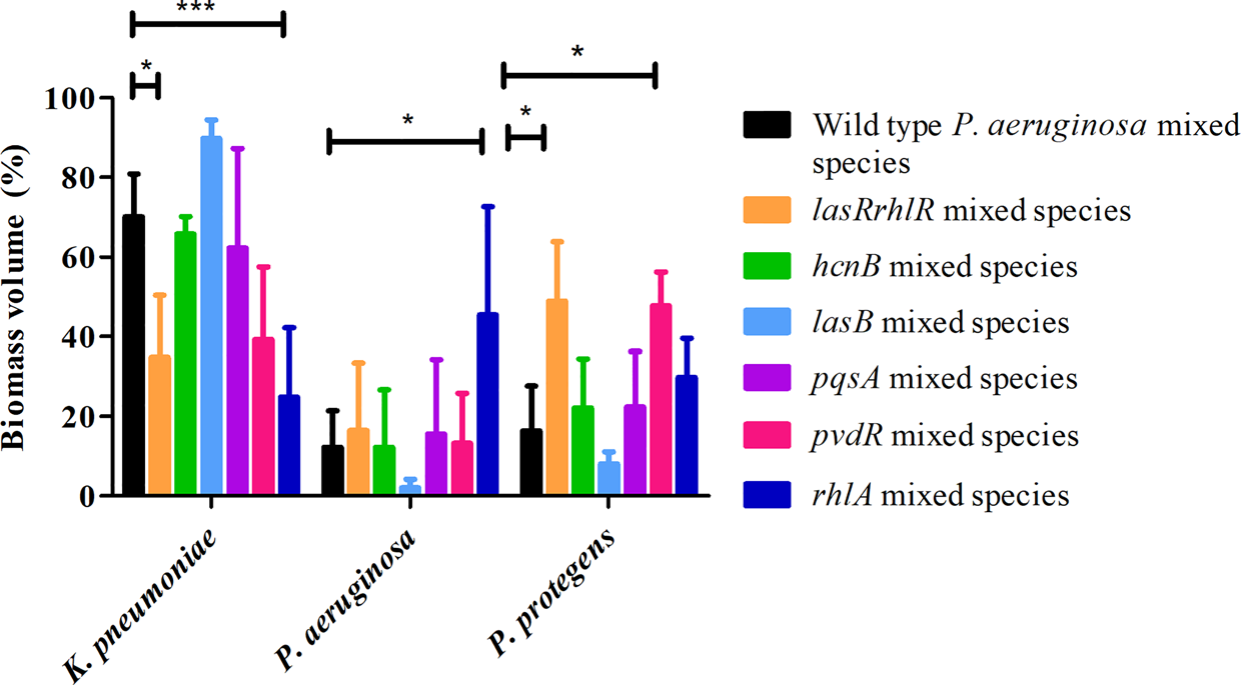
Proportions of mixed species biofilm containing mutants of QS regulated effectors. The proportions of the three species in the mixed species biofilm containing either the *P. aeruginosa* wild type, the QS mutant *lasRrhlR* or the QS target mutants *hcnB*, *lasB*, *pvdR*, *rhlA*, or *pqsA* after 3 days of cultivation. Bar charts depict the percentages of *K. pneumoniae*, *P. aeruginosa* and *P. protegens* in the wild type or the mutant mixed species biofilms. The proportions were derived by cultivating biofilms under flow cell conditions followed by quantitative image analysis of 2 independent biological replicates. Error bars represent standard deviations (n = 2). * and *** denote significant difference by two-way ANOVA followed by Tukey’s post-test (*P* < 0.05; *P* < 0.001).

When tested in triple species biofilms, for the communities containing mutants of *pvdR* and *rhlA*, the proportions of *K. pneumoniae*, 39.1 ± 18.3% and 24.7 ± 17.4%, respectively, were lower compared to biofilms with the wild type *P. aeruginosa* but only *rhlA* was significantly different (**Figure 6**). The proportion of *P. protegens* was significantly different only for the *pvdR* mutant mixed community (47.6 ± 8.5%). For the *lasRrhlR* community the proportions of *K. pneumoniae* and *P. protegens* were 34.7 ± 15.5% and 48.8 ± 14.8%, respectively, as expected. Interestingly, the mixed species biofilm community formed with the *rhlA* mutant had a significantly higher proportion of *P. aeruginosa* (45.4 ± 27.1%) compared to the wild type. Furthermore, the mixed species biofilm community formed with the *lasB* mutant had a significantly higher proportion of *K. pneumoniae* (89.8 ± 4.5%), and significantly lower proportion of *P. protegens* (8 ± 2.9%), when compared to wild type *P. aeruginosa* containing biofilm community (70 ± 10.8% and 16.2 ± 11.3%, respectively) (**Figure 6**). The mixed species biofilm community formed with the *hcnB* and *pqsA* mutant did not differ significantly from wild type *P. aeruginosa* containing biofilm in its composition. These results suggest that QS regulated genes do not contribute equally to the community composition and that none have the same magnitude of effect as the *lasrRrhlR* or *lasIrhlI* mutants. Thus, the QS effectors may have a cumulative effect in determining community composition.

## Discussion

In this work, we investigated the role of QS in interspecies interactions in a defined 3-species bacterial community during biofilm formation. We observed that the proportions of *K. pneumoniae* and *P. protegens* were dictated by the presence or absence of a functional *P. aeruginosa* QS system. We also show that there is no effect of deletion of the various QS genes on the relative fitness of the strains, further suggesting that it is the loss of QS function that drives the changes in community composition and structure. This effect was surprising as *P. aeruginosa* represents only 1-10% of the mature biofilm community, suggesting that even at a relatively low abundance, this species can significantly modulate community interactions. QS was shown to play a significant role in specifically modulating community composition between *P. aeruginosa* and *P. protegens*, with little to no effect on *K. pneumoniae*. We also showed that several downstream QS targets cumulatively mediate this competitive effect.

The complemented *lasRrhlR* mutant completely restored known QS regulated phenotypes, e.g. swarming, elastase activity and protease activity in *in vitro* assays. In addition, complementation also restored competitive advantage over *P. protegens* in growth assays on agar surface (**Figure 4**). While the relative abundances of the complemented strains were not significantly different from the wild type when grown in the mixed species biofilm, they were none the less slightly lower than for the wild type (**Figure S2**). The lower abundances achieved for the complemented strains might be due to higher and earlier expression of the QS genes from the multi-copy plasmids used for complementation. In addition to the amount of signal production, the level of signal receptor, LasR, production is known to be a critical factor in QS mediated gene expression linked to biofilm formation (Schuster et al., 2003; Rutherford and Bassler, 2012). This may in part be due to the expression of LasR and RhlR at late logarithmic and early stationary phase in planktonic cultures. Thus, given that the receptor genes were expressed on a multicopy plasmid, it is likely that the receptors were produced earlier in the complemented strain than normal. This could lead to the expression of QS regulated functions at a lower cell density by the complemented strain, which might consequently lead to its earlier detection by the competing bacterial species, *P. protegens*, likely leading to its removal and lower abundance. Therefore, optimal timing of expression of QS regulators LasR and RhlR might play an important role in the competition with *P. protegens* during early biofilm formation.

*K. pneumoniae* KP1 and *P. protegens* Pf-5 carry one and two *luxR* homologs, respectively, but they do not produce AHLs. It is possible that both *K. pneumoniae* and *P. protegens* in multispecies communities might sense and respond to foreign AHLs to regulate biofilm formation. This has previously been reported for multispecies biofilms formed by *P. aeruginosa* and *Burkholderia cepacia* in the lungs of CF patients (Van Delden and Iglewski, 1998; Huber et al., 2001; Riedel et al., 2001). Indeed, cross-species or cross-kingdom signal perception is the most commonly reported role of QS for communities. However, both of the *P. aeruginosa lasRrhlR* and *lasIrhlI* mutants show similar proportions in the 3-species community as do the individual QS effector mutants. This suggests that it is the *P. aeruginosa* QS regulon that mediates interaction with the other two species and not simply signal perception by those species through their LuxR homologs. However, it will be interesting to study whether AHLs directly influence biofilm formation by *P. protegens* and *K. pneumoniae* in future studies.

In dual species biofilms, there was no effect of QS on the relative abundance of either *K. pneumoniae* or *P. aeruginosa* (**Figure 2**) under the conditions used in this study. Also, the *K. pneumoniae* -* P. protegens* interaction appeared to be neutral, where both species were present in equal abundance (data not shown). However, QS provided a competitive advantage to *P. aeruginosa* when cultivated with *P. protegens* (**Figure 3**). Although there are several reports on the role of QS in laboratory dual species co-culture systems and it is predicted to influence more complex community dynamics, there is not much direct evidence for the latter, which has been shown here (Mazzola et al., 1992; Flemming and Wingender, 2010; Tan et al., 2014). Specifically, *P. aeruginosa* interaction with *B. cepacia* sp., *A. tumefaciens*, *S. aureus* and *B. cenocepacia* has been shown to be due to QS regulated antimicrobial production and AHL eavesdropping in planktonic cultures (Lewenza et al., 2002; An et al., 2006; Costello et al., 2014; Smalley et al., 2015). In *Agrobacterium tumefaciens-P. aeruginosa* dual species biofilms, the wild type *P. aeruginosa* completely blanketed *A. tumefaciens* and progressively excluded *A. tumefaciens* from the biofilm while a *P. aeruginosa* QS mutant did not. This was controlled by antimicrobial production and swarming (An et al., 2006). We propose that *P. aeruginosa* -* P. protegens* interactions in dual species biofilms is a two-way interaction involving functions from both bacteria which provide them competitive advantage and results in mutual exclusion. A number of QS regulated *P. aeruginosa* products such as rhamnolipids, hydrogen cyanide, pyocyanin and pyoverdine are known to be important for competition with other bacteria; these effects are likely to be multifactorial with some antimicrobials having a greater effect (Smalley et al., 2015). Similarly, *P. protegens* is also known to produce a cocktail of both antifungal and antibacterial secondary metabolites including 2,4-diacetylphloroglucinol, pyoluteorin, pyrrolnitrin, extracellular protease, hydrogen cyanide and siderophores pyochelin and pyoverdine (Paulsen et al., 2005). It has been proposed that the natural function of multi-drug resistance efflux pumps in *P. aeruginosa* could be to excrete a particular class of secondary metabolite (Poole, 1994). It is thus possible that QS regulated efflux systems also protects *P. aeruginosa* against one or more antimicrobials produced by *P. protegens* and future work will address these mechanisms.

Our results with QS and target mutants revealed that most of the mutants except *aprD* were proficient for biofilm formation in batch cultivation assays (**Figure S4**). Previous reports indicate that *P. aeruginosa* QS is required for biofilm formation. Specifically, the Las system has been shown to control progression from reversible to irreversible attachment whereas the Rhl system is responsible for biofilm maturation (Jaffar-Bandjee et al., 1995; Davies et al., 1998). However, in our experiments, monospecies biofilms, formed by *lasIrhlI* and *lasRrhlR* mutants under flow-cell conditions did not show any difference compared to *P. aeruginosa* wild type in terms of biovolume and thickness (**Figure S8**). Moreover, among the QS target mutants, *pqsA*, *pqsE* and *rhlA* have also shown to be defective for biofilm maturation (Yang et al., 2009; Darveau et al., 2012; Hajishengallis et al., 2012). The effects of Pqs system on biofilm structure was shown to be iron dependent. Although *rhlA* mutant has been shown to form flat biofilms lacking heterogeneity of wild type biofilms in the flow-cell system, in the same study it has been reported that in microtiter dish assays the *rhlA* mutant is more proficient at early colonization (up to 24 h) than is the wild type (Darveau et al., 2012). Active iron uptake has been shown to be important for *P. aeruginosa* biofilm architecture as *pvdS* and *pvdA* mutants form thin biofilms (Banin et al., 2005; Yang et al., 2009). However, *pvdA* and *pvdE* mutants form nearly normal biofilms in microtiter dish assays (Kang and Kirienko, 2017). Our experiments to test biofilm formation by QS target mutants was carried out under batch culture conditions. As described above, there are differences in the medium and carbon source/s, cultivation temperature and cultivation system between the previous studies with these mutants and the work we have presented here; this might account for the observed biofilm formation phenotypes. For example, Davies et al. (Davies et al., 1998) showed that the QS defect in biofilm formation by *P. aeruginosa* could be overcome by growing the mutant in complex (LB) rather than defined media.

In order to narrow down the processes involved in competition, we investigated interactions between *P. aeruginosa* and *P. protegens* in dual and 3-species biofilms where the wild type *P. aeruginosa* was substituted by mutants of several QS regulated genes, including *rhlA* and *pvdR*. *rhlA* mutant was significantly less competitive than wild type in dual species community with *P. protegens* (**Figure 5**). Interestingly, when wild type *P. aeruginosa* was substituted by the *rhlA* mutant in the 3-species community, there was a significant increase in the biomass of the *rhlA* mutant (**Figure 6**). Mutants in *rhlA* are defective for the production of the biosurfactant rhamnolipid which promotes motility and affects the architecture of monospecies *P. aeruginosa* biofilms (Davey et al., 2003). Rhamnolipids might affect both cell-cell and cell-surface interactions (Neu, 1996). In fact, in the wild type, when rhamnolipids are produced, they might prevent self-colonization as well as colonization by other species. This inhibition is lacking in the *rhlA* mutant community. Only *pvdR* showed trends of alteration of both *K. pneumoniae* and *P. protegens* similar to the QS mutants in 3-species biofilms, although in dual species *pvdR* community this difference was not significant. PvdR is involved pyoverdine transport and recycling in *P. aeruginosa* and its mutants accumulate pyoverdine in the periplasmic space and are unable to acquire ferric iron efficiently *via* pyoverdine pathway (Imperi et al., 2009). This suggests that optimal iron uptake and possibly use of heterologous siderophores might be important for maintaining the 3-species community composition as fluorescent Pseudomonads are known to produce multiple pyoverdines (Cornelis and Matthijs, 2002). Together, these results suggest that QS regulated effectors affect the community composition in different ways. Specifically, we found that reduction of *K. pneumoniae* biomass was not always linked to increase in *P. protegens* biomass in the 3-species biofilm. It is also interesting that QS did not alter the relationship between *P. aeruginosa* and *K. pneumoniae* despite the clear reduction in the latter when the QS mutants of *P. aeruginosa* are present in the three species community. Further work will be needed to elucidate how QS effectors operate in the three species community to control composition. Similarly, the mechanisms of competition mediated by secondary metabolites of *P. protegens* and their role in biofilms require further investigation and are beyond the scope of the present study.

The least abundant bacterium in this 3-species biofilm community, *P. aeruginosa*, determined the proportions of the other two species. This finding shows an effect of QS on non-clonal cells even when present in low abundance in a polymicrobial community. It has been similarly reported that *Microbacterium oxydans*, the least abundant species, determined the spatial organization of others in a four-species community (Liu et al., 2017). Additionally, *P. gingivalis*, which is present in relatively low abundance, is responsible for triggering changes in both amount and composition of oral commensal microbiota (Hajishengallis et al., 2011). These bacteria are examples of keystone species in a community (Hajishengallis and Lamont, 2016). Low abundant species in communities are often overlooked due to the limitation of omics approaches in delineating their role in these biofilms. Despite some of those limitations, it is none the less possible to identify potential keystone species in complex communities, such as wastewater systems, through network analysis. Indeed, such network analyses have been extended to functional genes within sludge communities to identify those genes, termed keystone genes, that play essential, linking roles, between community members and presumably sludge function (Roume et al., 2015). The role of low-abundance keystone pathogens that promote the formation and stabilization of dysbiotic and disease-inducing communities in the human gut have been reported (Hajishengallis et al., 2012). There are also keystone organisms that act as stabilizers that kill opportunistic pathogens but not the indigenous gut bacteria (Stecher et al., 2013). For example, it has been shown that *P. aeruginosa* senses peptidoglycan and diffusible signals produced by other bacteria to modify community composition and persistence in *P. aeruginosa*-dominated disease conditions (Twomey et al., 2012; Korgaonkar et al., 2013). The role played by low abundance bacteria producing these signals in shaping community-level changes would be consistent with their position as keystone bacteria. Our findings clearly demonstrate that a bacterium does not need to be the most abundant organism in the biofilm to be important for controlling the community structure and composition of bacterial species. In this way, *P. aeruginosa* serves as a keystone species in this biofilm community. The results presented here also demonstrate how a genetically amenable system can be well suited to test hypotheses about the roles and mechanisms of keystone species in mixed species communities.

Of the three species used in this study, *P. aeruginosa* and *K. pneumoniae* are opportunistic human pathogens known to occur together in human-associated infections such as respiratory tract, chronic wound and urinary tract infections (Harmsen et al., 2010; Rezaei et al., 2011; Magill et al., 2014). Both bacteria adapt to airways and other biotic surfaces through biofilm formation which contributes to the success of these pathogens (Riquelme et al., 2018). These bacteria often occur with other bacteria in polymicrobial interactions associated with human disease and some of these interactions have been studied (Peters et al., 2012). *P. protegens*, predominantly studied as a plant-protecting bacterium, is not generally considered a human pathogen although bacteria of the *P. fluorescens* complex have been isolated in clinical samples from mouth, stomach and lungs (Paulsen et al., 2005; Scales et al., 2014). Colonization studies of roots and biofilm formation by *P. protegens* have been reported but interaction with other members of rhizosphere communities is less studied. Most of the community studies use a top-down approach to interrogate the properties of community dynamics and function. However, these studies have yielded less information regarding specific mechanisms, interactions and community assembly. Understanding the different 2-species interactions in our mixed-species community is an advantage for future studies with increasing community complexity made up of bacterial species having either clinical or environmental relevance.

In conclusion, *P. aeruginosa* QS is an important modulator of the composition and structure of the 3-species biofilm community. In this system, *P. aeruginosa* functions as a keystone species that modulates the relative abundances of the species within its local environment. Future studies need to focus on the mechanisms behind changes in the proportions of both *K. pneumoniae* and *P. protegens* in the 3-species biofilm community. Competitive interactions between two members of this community, *P. aeruginosa* and *P. protegens*, appear to be responsible for this phenotype. Therefore, further studies on *P. aeruginosa* QS regulated functions and *P. protegens* secondary metabolites might help us to understand principles of antagonistic and competitive interactions within a developing biofilm.

## Data Availability Statement

The original contributions presented in the study are publicly available. This data can be found here: https://doi.org/10.21979/N9/GV37YR.

## Author Contributions

SS, SK, and SR conceived and planned the experiments. SS and MM carried out the experiments. SS, SB, MM, SK, and SR contributed to the interpretation of the results. SS, SK, and SR wrote the manuscript. All authors contributed to discussions on the manuscript. All authors contributed to the article and approved the submitted version.

## Funding

The authors would like to acknowledge the financial support from the National Research Foundation and Ministry of Education Singapore under its Research Centre of Excellence Program. The research was further supported by a grant from the Singapore Ministry of Education (MOE2019-T2-1-050).

## Conflict of Interest

The authors declare that the research was conducted in the absence of any commercial or financial relationships that could be construed as a potential conflict of interest.

## Supplementary Material

Supplementary Figure 1 QS regulated phenotypes of *P. aeruginosa* wild type, *lasRrhlR* mutant and the complemented strain. **(A)** Swarming motility of indicated strains on 0.5% swarm agar. Plates were incubated at 37°C for 16 h. Bars represent average diameter of the swarm area. **(B)** Protease activity of indicated strains on 1% skimmed milk containing LB agar plates. Plates were incubated at 37°C for 16 h. Bars represent average diameter of the zone of clearance around the colonies. **(C)** Elastase activity of indicated strains measured by mixing 100 µl of culture supernatant with 900 µl of Elastin-Congo Red containing buffer and incubating overnight at 37°C. Elastase activity was calculated by measuring the absorbance (OD_495_) of the supernatant. Bars represent OD_495_ values obtained after subtracting the values of medium control from the sample values. The data are averages from three independent biological replicates. **** denotes significant difference by One-way ANOVA followed by Tukey’s post-test (*P*<0.0001). 1, wild type *P. aeruginosa*; 2, *lasRrhlR*; 3, *lasRrhlR/*pUCP22notI+R2.

Supplementary Figure 2 Proportions of *P. aeruginosa* wild type, *lasRrhlR* mutant and the complemented strain in dual-species biofilms. The proportions of the two species in the dual-species biofilm containing either the *P. aeruginosa* wild type, the QS mutant *lasRrhlR* or the complemented strain after 3 d of cultivation. Bar charts depict the percentages of *P. aeruginosa* and *P. protegens* in the wild type, the mutant or the complemented dual-species biofilms. The proportions were derived by cultivating biofilms under flow cell conditions followed by quantitative image analysis of two independent biological replicates. Error bars represent standard deviations (n=2). * denotes significant difference by Two-way ANOVA followed by Tukey’s post-test (*P*<0.05).

Supplementary Figure 3 Growth curves of wild type and QS effector mutants of *P. aeruginosa* in M9 minimal medium containing glucose and casamino acids. *P. aeruginosa* wild type and different QS effector mutants were incubated at room temperature in 96 well microtiter plates and absorbance (OD_600_) recorded at regular intervals. Data shown are average of three independent biological replicates.

Supplementary Figure 4 Biofilm formation of wild type and QS effector mutants of *P. aeruginosa* in M9 minimal medium containing glucose and casamino acids. *P. aeruginosa* WT and different QS effector mutants were incubated at room temperature in 24 well microtiter plates and the amount of biofilm formed quantified as absorbance (OD_570_) after crystal violet staining. Data shown are average of 3 biological replicates.

Supplementary Figure 5 Fitness assays for wild type and QS effector mutants of *P. aeruginosa* in the presence of *P. protegens* in planktonic cultures. Equal volumes of *P. aeruginosa* wild type or QS effector mutants were mixed with *P. protegens* and allowed to grow for 24 h at room temperature. Bacteria were enumerated at zero and 24 h. Fitness was plotted as Log[Fold change of mutant/fold change of wild type]. Data shown are average of three independent biological replicates.

Supplementary Figure 6
*Pseudomonas aeruginosa* mutants are not affected in competition with *K. pneumoniae*. *K. pneumoniae* was spotted on a lawn of *P. aeruginosa* wild type, QS mutants or complemented strains on LB agar plate. This panel shows the plate phenotype on day1 and day2.

Supplementary Figure 7
*Pseudomonas aeruginosa* QS target mutants are not affected in competition with *P. protegens*. *P. protegens* was spotted on a lawn of *P. aeruginosa* wild type or QS target mutants on LB agar plate. This panel shows the plate phenotype on day1 and day2.

Supplementary Figure 8 Proportions of the *P. aeruginosa* monospecies biofilm. The biovolume per unit area in the monospecies biofilm containing either the *P. aeruginosa* wild type or the QS mutants (*lasIrhlI* or *lasrRrhlR*) after 3 d of cultivation. Bar charts depict the biovolume per unit area of *P. aeruginosa* wild type or QS mutant monospecies biofilm. The values were derived by cultivating biofilms under flow cell conditions followed by quantitative image analysis of two independent biological replicates.

## References

[B1] Abdel-Mawgoud A. M. Lepine F. Deziel E. (2010). Rhamnolipids: diversity of structures, microbial origins and roles. Appl. Microbiol. Biotechnol. 86 (5), 1323–1336. 10.1007/s00253-010-2498-2 20336292 PMC2854365

[B2] An D. Danhorn T. Fuqua C. Parsek M. R. (2006). Quorum sensing and motility mediate interactions between Pseudomonas aeruginosa and Agrobacterium tumefaciens in biofilm cocultures. Proc. Natl. Acad. Sci. U. S. A. 103 (10), 3828–3833. 10.1073/pnas.0511323103 16537456 PMC1533783

[B3] An S. Wu J. Zhang L. H. (2010). Modulation of Pseudomonas aeruginosa biofilm dispersal by a cyclic-Di-GMP phosphodiesterase with a putative hypoxia-sensing domain. Appl. Environ. Microbiol. 76 (24), 8160–8173. 10.1128/AEM.01233-10 20971871 PMC3008239

[B4] Anand A. A. Vennison S. J. Sankar S. G. Prabhu D. I. Vasan P. T. Raghuraman T.. (2010). Isolation and characterization of bacteria from the gut of Bombyx mori that degrade cellulose, xylan, pectin and starch and their impact on digestion. J. Insect Sci. 10:107. 10.1673/031.010.10701 20874394 PMC3016902

[B5] Banin E. Vasil M. L. Greenberg E. P. (2005). Iron and Pseudomonas aeruginosa biofilm formation. Proc. Natl. Acad. Sci. U.S.A. 102 (31), 11076–11081. 10.1073/pnas.0504266102 16043697 PMC1182440

[B6] Brown S. A. Whiteley M. (2007). A novel exclusion mechanism for carbon resource partitioning in Aggregatibacter actinomycetemcomitans. J. Bacteriol 189 (17), 6407–6414. 10.1128/JB.00554-07 17586632 PMC1951915

[B7] Chang C. Y. Krishnan T. Wang H. Chen Y. Yin W. F. Chong Y. M.. (2014). Non-antibiotic quorum sensing inhibitors acting against N-acyl homoserine lactone synthase as druggable target. Sci. Rep. 4, 7245. 10.1038/srep07245 25430794 PMC4246208

[B8] Chazal P. M. (1995). Pollution of modern metalworking fluids containing biocides by pathogenic bacteria in France. Reexamination of chemical treatments accuracy. Eur. J. Epidemiol. 11 (1), 1–7. 10.1007/BF01719939 7489767

[B9] Choi K. H. Schweizer H. P. (2005). An improved method for rapid generation of unmarked Pseudomonas aeruginosa deletion mutants. BMC Microbiol. 5:30. 10.1186/1471-2180-5-30 15907219 PMC1173109

[B10] Choi K. H. Schweizer H. P. (2006). mini-Tn7 insertion in bacteria with single attTn7 sites: example Pseudomonas aeruginosa. Nat. Protoc. 1 (1), 153–161. 10.1038/nprot.2006.24 17406227

[B11] Choi K. H. Gaynor J. B. White K. G. Lopez C. Bosio C. M. Karkhoff-Schweizer R. R.. (2005). A Tn7-based broad-range bacterial cloning and expression system. Nat. Methods 2 (6), 443–448. 10.1038/nmeth765 15908923

[B12] Clark D. J. Maaløe O. (1967). DNA replication and the division cycle in Escherichia coli. J. Mol. Biol. 23 (1), 99 – 112. 10.1016/S0022-2836(67)80070-6

[B13] Cornelis P. Matthijs S. (2002). Diversity of siderophore-mediated iron uptake systems in fluorescent pseudomonads: not only pyoverdines. Environ. Microbiol. 4 (12), 787–798. 10.1046/j.1462-2920.2002.00369.x 12534462

[B14] Costello A. Reen F. J. O’Gara F. Callaghan M. McClean S. (2014). Inhibition of co-colonizing cystic fibrosis-associated pathogens by Pseudomonas aeruginosa and Burkholderia multivorans. Microbiology (Reading) 160 (Pt 7), 1474–1487. 10.1099/mic.0.074203-0 24790091

[B15] Darveau R. P. Hajishengallis G. Curtis M. A. (2012). Porphyromonas gingivalis as a potential community activist for disease. J. Dent. Res. 91 (9), 816–820. 10.1177/0022034512453589 22772362 PMC3420389

[B16] Davey M. E. O’Toole G A. (2000). Microbial biofilms: from ecology to molecular genetics. Microbiol. Mol. Biol. Rev. 64 (4), 847–867. 10.1128/mmbr.64.4.847-867.2000 11104821 PMC99016

[B17] Davey M. E. Caiazza N. C. O’Toole G. A. (2003). Rhamnolipid surfactant production affects biofilm architecture in Pseudomonas aeruginosa PAO1. J. Bacteriol 185 (3), 1027–1036. 10.1128/jb.185.3.1027-1036.2003 12533479 PMC142794

[B18] Davies D. G. Parsek M. R. Pearson J. P. Iglewski B. H. Costerton J. W. Greenberg E. P. (1998). The involvement of cell-to-cell signals in the development of a bacterial biofilm. Science 280 (5361), 295–298. 10.1126/science.280.5361.295 9535661

[B19] Flemming H. C. Wingender J. (2010). The biofilm matrix. Nat. Rev. Microbiol. 8 (9), 623–633. 10.1038/nrmicro2415 20676145

[B20] Flemming H. C. Wingender J. Szewzyk U. Steinberg P. Rice S. A. Kjelleberg S. (2016). Biofilms: an emergent form of bacterial life. Nat. Rev. Microbiol. 14 (9), 563–575. 10.1038/nrmicro.2016.94 27510863

[B21] Gambello M. J. Iglewski B. H. (1991). Cloning and characterization of the Pseudomonas aeruginosa lasR gene, a transcriptional activator of elastase expression. J. Bacteriol 173 (9), 3000–3009. 10.1128/jb.173.9.3000-3009.1991 1902216 PMC207884

[B22] Hajishengallis G. Lamont R. J. (2016). Dancing with the Stars: How Choreographed Bacterial Interactions Dictate Nososymbiocity and Give Rise to Keystone Pathogens, Accessory Pathogens, and Pathobionts. Trends Microbiol. 24 (6), 477–489. 10.1016/j.tim.2016.02.010 26968354 PMC4874887

[B23] Hajishengallis G. Liang S. Payne M. A. Hashim A. Jotwani R. Eskan M. A.. (2011). Low-abundance biofilm species orchestrates inflammatory periodontal disease through the commensal microbiota and complement. Cell Host Microbe 10 (5), 497–506. 10.1016/j.chom.2011.10.006 22036469 PMC3221781

[B24] Hajishengallis G. Darveau R. P. Curtis M. A. (2012). The keystone-pathogen hypothesis. Nat. Rev. Microbiol. 10 (10), 717–725. 10.1038/nrmicro2873 22941505 PMC3498498

[B25] Harmsen M. Yang L. Pamp S. J. Tolker-Nielsen T. (2010). An update on Pseudomonas aeruginosa biofilm formation, tolerance, and dispersal. FEMS Immunol. Med. Microbiol. 59 (3), 253–268. 10.1111/j.1574-695X.2010.00690.x 20497222

[B26] Heydorn A. Ersboll B. K. Hentzer M. Parsek M. R. Givskov M. Molin S. (2000a). Experimental reproducibility in flow-chamber biofilms. Microbiology (Reading) 146 ( Pt 10), 2409–2415. 10.1099/00221287-146-10-2409 11021917

[B27] Heydorn A. Nielsen A. T. Hentzer M. Sternberg C. Givskov M. Ersboll B. K.. (2000b). Quantification of biofilm structures by the novel computer program COMSTAT. Microbiology (Reading) 146 ( Pt 10), 2395–2407. 10.1099/00221287-146-10-2395 11021916

[B28] Hoang T. T. Karkhoff-Schweizer R. R. Kutchma A. J. Schweizer H. P. (1998). A broad-host-range Flp-FRT recombination system for site-specific excision of chromosomally-located DNA sequences: application for isolation of unmarked Pseudomonas aeruginosa mutants. Gene 212 (1), 77–86. 10.1016/s0378-1119(98)00130-9 9661666

[B29] Hosni T. Moretti C. Devescovi G. Suarez-Moreno Z. R. Fatmi M. B. Guarnaccia C.. (2011). Sharing of quorum-sensing signals and role of interspecies communities in a bacterial plant disease. ISME J. 5 (12), 1857–1870. 10.1038/ismej.2011.65 21677694 PMC3223305

[B30] Huber B. Riedel K. Hentzer M. Heydorn A. Gotschlich A. Givskov M.. (2001). The cep quorum-sensing system of Burkholderia cepacia H111 controls biofilm formation and swarming motility. Microbiology 147 (Pt 9), 2517–2528. 10.1099/00221287-147-9-2517 11535791

[B31] Imperi F. Tiburzi F. Visca P. (2009). Molecular basis of pyoverdine siderophore recycling in Pseudomonas aeruginosa. Proc. Natl. Acad. Sci. U.S.A. 106 (48), 20440–20445. 10.1073/pnas.0908760106 19906986 PMC2787144

[B32] Jackson G. Beyenal H. Rees W. M. Lewandowski Z. (2001). Growing reproducible biofilms with respect to structure and viable cell counts. J. Microbiol. Methods 47 (1), 1–10. 10.1016/s0167-7012(01)00280-9 11566221

[B33] Jaffar-Bandjee M. C. Lazdunski A. Bally M. Carrere J. Chazalette J. P. Galabert C. (1995). Production of elastase, exotoxin A, and alkaline protease in sputa during pulmonary exacerbation of cystic fibrosis in patients chronically infected by Pseudomonas aeruginosa. J. Clin. Microbiol. 33 (4), 924–929. 10.1128/JCM.33.4.924-929.1995 7790462 PMC228069

[B34] Kang D. Kirienko N. V. (2017). High-Throughput Genetic Screen Reveals that Early Attachment and Biofilm Formation Are Necessary for Full Pyoverdine Production by Pseudomonas aeruginosa. Front. Microbiol. 8, 1707. 10.3389/fmicb.2017.01707 28928729 PMC5591869

[B35] Kessler B. de Lorenzo V. Timmis K. N. (1992). A general system to integrate lacZ fusions into the chromosomes of gram-negative eubacteria: regulation of the Pm promoter of the TOL plasmid studied with all controlling elements in monocopy. Mol. Gen. Genet. 233 (1-2), 293–301. 10.1007/BF00587591 1318499

[B36] Kommerein N. Doll K. Stumpp N. S. Stiesch M. (2018). Development and characterization of an oral multispecies biofilm implant flow chamber model. PloS One 13 (5), e0196967. 10.1371/journal.pone.0196967 29771975 PMC5957423

[B37] Korgaonkar A. Trivedi U. Rumbaugh K. P. Whiteley M. (2013). Community surveillance enhances Pseudomonas aeruginosa virulence during polymicrobial infection. Proc. Natl. Acad. Sci. U. S. A. 110 (3), 1059–1064. 10.1073/pnas.1214550110 23277552 PMC3549110

[B38] Lee J. Zhang L. (2015). The hierarchy quorum sensing network in Pseudomonas aeruginosa. Protein Cell 6 (1), 26–41. 10.1007/s13238-014-0100-x 25249263 PMC4286720

[B39] Lee J. Wu J. Deng Y. Wang J. Wang C. Wang J.. (2013). A cell-cell communication signal integrates quorum sensing and stress response. Nat. Chem. Biol. 9 (5), 339–343. 10.1038/nchembio.1225 23542643

[B40] Lee K. W. Periasamy S. Mukherjee M. Xie C. Kjelleberg S. Rice S. A. (2014). Biofilm development and enhanced stress resistance of a model, mixed-species community biofilm. ISME J. 8 (4), 894–907. 10.1038/ismej.2013.194 24152718 PMC3960537

[B41] Lesic B. Starkey M. He J. Hazan R. Rahme L. G. (2009). Quorum sensing differentially regulates Pseudomonas aeruginosa type VI secretion locus I and homologous loci II and III, which are required for pathogenesis. Microbiology 155 (Pt 9), 2845–2855. 10.1099/mic.0.029082-0 19497948 PMC2888175

[B42] Lewenza S. Visser M. B. Sokol P. A. (2002). Interspecies communication between Burkholderia cepacia and Pseudomonas aeruginosa. Can. J. Microbiol. 48 (8), 707–716. 10.1139/w02-068 12381027

[B43] Lim C. K. Hassan K. A. Penesyan A. Loper J. E. Paulsen I. T. (2013). The effect of zinc limitation on the transcriptome of Pseudomonas protegens Pf-5. Environ. Microbiol. 15 (3), 702–715. 10.1111/j.1462-2920.2012.02849.x 22900619

[B44] Liu W. Russel J. Roder H. L. Madsen J. S. Burmolle M. Sorensen S. J. (2017). Low-abundant species facilitates specific spatial organization that promotes multispecies biofilm formation. Environ. Microbiol. 19 (7), 2893–2905. 10.1111/1462-2920.13816 28618083

[B45] Magill S. S. Edwards J. R. Bamberg W. Beldavs Z. G. Dumyati G. Kainer M. A.. (2014). Multistate point-prevalence survey of health care-associated infections. N. Engl. J. Med. 370 (13), 1198–1208. 10.1056/NEJMoa1306801 24670166 PMC4648343

[B46] Mazzola M. Cook R. J. Thomashow L. S. Weller D. M. Pierson L. S. (1992). Contribution of phenazine antibiotic biosynthesis to the ecological competence of fluorescent pseudomonads in soil habitats. Appl. Environ. Microbiol. 58 (8), 2616–2624. 10.1128/AEM.58.8.2616-2624.1992 1514808 PMC195829

[B47] Moons P. Van Houdt R. Aertsen A. Vanoirbeek K. Engelborghs Y. Michiels C. W. (2006). Role of Quorum Sensing and Antimicrobial Component Production by *Serratia plymuthica* in Formation of Biofilms, Including Mixed Biofilms with *Escherichia coli* . Appl. Environ. Microbiol. 72 (11), 7294–7300. 10.1128/AEM.01708-06 16997989 PMC1636202

[B48] Neu T. R. (1996). Significance of bacterial surface-active compounds in interaction of bacteria with interfaces. Microbiol. Rev. 60 (1), 151–166. 10.1128/MR.60.1.151-166.1996 8852899 PMC239423

[B49] Ochsner U. A. Reiser J. (1995). Autoinducer-mediated regulation of rhamnolipid biosurfactant synthesis in Pseudomonas aeruginosa. Proc. Natl. Acad. Sci. U. S. A. 92 (14), 6424–6428. 10.1073/pnas.92.14.6424 7604006 PMC41530

[B50] Pamp S. J. Tolker-Nielsen T. (2007). Multiple roles of biosurfactants in structural biofilm development by Pseudomonas aeruginosa. J. Bacteriol 189 (6), 2531–2539. 10.1128/JB.01515-06 17220224 PMC1899385

[B51] Paulsen I. T. Press C. M. Ravel J. Kobayashi D. Y. Myers G. S. Mavrodi D. V.. (2005). Complete genome sequence of the plant commensal Pseudomonas fluorescens Pf-5. Nat. Biotechnol. 23 (7), 873–878. 10.1038/nbt1110 15980861 PMC7416659

[B52] Pearson J. P. Passador L. Iglewski B. H. Greenberg E. P. (1995). A second N-acylhomoserine lactone signal produced by Pseudomonas aeruginosa. Proc. Natl. Acad. Sci. U. S. A. 92 (5), 1490–1494. 10.1073/pnas.92.5.1490 7878006 PMC42545

[B53] Peters B. M. Jabra-Rizk M. A. O’May G. A. Costerton J. W. Shirtliff M. E. (2012). Polymicrobial interactions: impact on pathogenesis and human disease. Clin. Microbiol. Rev. 25 (1), 193–213. 10.1128/CMR.00013-11 22232376 PMC3255964

[B54] Poole K. (1994). Bacterial multidrug resistance–emphasis on efflux mechanisms and Pseudomonas aeruginosa. J. Antimicrob. Chemother. 34 (4), 453–456. 10.1093/jac/34.4.453 7868400

[B55] Rahim R. Ochsner U. A. Olvera C. Graninger M. Messner P. Lam J. S.. (2001). Cloning and functional characterization of the Pseudomonas aeruginosa rhlC gene that encodes rhamnosyltransferase 2, an enzyme responsible for di-rhamnolipid biosynthesis. Mol. Microbiol. 40 (3), 708–718. 10.1046/j.1365-2958.2001.02420.x 11359576

[B56] Ramette A. Frapolli M. Fischer-Le Saux M. Gruffaz C. Meyer J. M. Defago G.. (2011). Pseudomonas protegens sp. nov., widespread plant-protecting bacteria producing the biocontrol compounds 2,4-diacetylphloroglucinol and pyoluteorin. Syst. Appl. Microbiol. 34 (3), 180–188. 10.1016/j.syapm.2010.10.005 21392918

[B57] Rezaei E. Safari H. Naderinasab M. Aliakbarian H. (2011). Common pathogens in burn wound and changes in their drug sensitivity. Burns 37 (5), 805–807. 10.1016/j.burns.2011.01.019 21388742

[B58] Riedel K. Hentzer M. Geisenberger O. Huber B. Steidle A. Wu H.. (2001). N-acylhomoserine-lactone-mediated communication between Pseudomonas aeruginosa and Burkholderia cepacia in mixed biofilms. Microbiology 147 (Pt 12), 3249–3262. 10.1099/00221287-147-12-3249 11739757

[B59] Riquelme S. A. Ahn D. Prince A. (2018). Pseudomonas aeruginosa and Klebsiella pneumoniae Adaptation to Innate Immune Clearance Mechanisms in the Lung. J. Innate Immun. 10 (5-6), 442–454. 10.1159/000487515 29617698 PMC6785651

[B60] Roume H. Heintz-Buschart A. Muller E. E. L. May P. Satagopam V. P. Laczny C. C.. (2015). Comparative integrated omics: identification of key functionalities in microbial community-wide metabolic networks. NPJ Biofilms Microbiomes 1 (1), 15007. 10.1038/npjbiofilms.2015.7 28721231 PMC5515219

[B61] Rutherford S. T. Bassler B. L. (2012). Bacterial quorum sensing: its role in virulence and possibilities for its control. Cold Spring Harb. Perspect. Med. 2 (11), a012427. 10.1101/cshperspect.a012427 23125205 PMC3543102

[B62] Scales B. S. Dickson R. P. LiPuma J. J. Huffnagle G. B. (2014). Microbiology, genomics, and clinical significance of the Pseudomonas fluorescens species complex, an unappreciated colonizer of humans. Clin. Microbiol. Rev. 27 (4), 927–948. 10.1128/CMR.00044-14 25278578 PMC4187640

[B63] Schleheck D. Barraud N. Klebensberger J. Webb J. S. McDougald D. Rice S. A.. (2009). Pseudomonas aeruginosa PAO1 preferentially grows as aggregates in liquid batch cultures and disperses upon starvation. PloS One 4 (5), e5513. 10.1371/journal.pone.0005513 19436737 PMC2677461

[B64] Schuster M. Lostroh C. P. Ogi T. Greenberg E. P. (2003). Identification, timing, and signal specificity of Pseudomonas aeruginosa quorum-controlled genes: a transcriptome analysis. J. Bacteriol 185 (7), 2066–2079. 10.1128/jb.185.7.2066-2079.2003 12644476 PMC151497

[B65] Simon R. Priefer U. Pühler A. (1983). A Broad Host Range Mobilization System for In Vivo Genetic Engineering: Transposon Mutagenesis in Gram Negative Bacteria. Biotechnology 1 (9), 784–791. 10.1038/nbt1183-784

[B66] Smalley N. E. An D. Parsek M. R. Chandler J. R. Dandekar A. A. (2015). Quorum Sensing Protects Pseudomonas aeruginosa against Cheating by Other Species in a Laboratory Coculture Model. J. Bacteriol 197 (19), 3154–3159. 10.1128/JB.00482-15 26195596 PMC4560282

[B67] Stecher B. Maier L. Hardt W. D. (2013). ‘Blooming’ in the gut: how dysbiosis might contribute to pathogen evolution. Nat. Rev. Microbiol. 11 (4), 277–284. 10.1038/nrmicro2989 23474681

[B68] Stoodley P. Wilson S. Hall-Stoodley L. Boyle J. D. Lappin-Scott H. M. Costerton J. W. (2001). Growth and detachment of cell clusters from mature mixed-species biofilms. Appl. Environ. Microbiol. 67 (12), 5608–5613. 10.1128/AEM.67.12.5608-5613.2001 11722913 PMC93350

[B69] Tan C. H. Koh K. S. Xie C. Tay M. Zhou Y. Williams R.. (2014). The role of quorum sensing signalling in EPS production and the assembly of a sludge community into aerobic granules. ISME J. 8 (6), 1186–1197. 10.1038/ismej.2013.240 24430488 PMC4030234

[B70] Twomey K. B. O’Connell O. J. McCarthy Y. Dow J. M. O’Toole G. A. Plant B. J.. (2012). Bacterial cis-2-unsaturated fatty acids found in the cystic fibrosis airway modulate virulence and persistence of Pseudomonas aeruginosa. ISME J. 6 (5), 939–950. 10.1038/ismej.2011.167 22134647 PMC3329111

[B71] Van Delden C. Iglewski B. H. (1998). Cell-to-cell signaling and Pseudomonas aeruginosa infections. Emerg. Infect. Dis. 4 (4), 551–560. 10.3201/eid0404.980405 9866731 PMC2640238

[B72] West S. E. Schweizer H. P. Dall C. Sample A. K. Runyen-Janecky L. J. (1994). Construction of improved Escherichia-Pseudomonas shuttle vectors derived from pUC18/19 and sequence of the region required for their replication in Pseudomonas aeruginosa. Gene 148 (1), 81–86. 10.1016/0378-1119(94)90237-2 7926843

[B73] Yang L. Nilsson M. Gjermansen M. Givskov M. Tolker-Nielsen T. (2009). Pyoverdine and PQS mediated subpopulation interactions involved in Pseudomonas aeruginosa biofilm formation. Mol. Microbiol. 74 (6), 1380–1392. 10.1111/j.1365-2958.2009.06934.x 19889094

[B74] Yin H. Deng Y. Wang H. Liu W. Zhuang X. Chu W. (2015). Tea polyphenols as an antivirulence compound Disrupt Quorum-Sensing Regulated Pathogenicity of Pseudomonas aeruginosa. Sci. Rep. 5, 16158. 10.1038/srep16158 26548447 PMC4637895

