# Supplementary figures and images for: N-Acyl Homoserine Lactone-Mediated Quorum Sensing Regulates Species Interactions in Multispecies Biofilm Communities
Source: Front Cell Infect Microbiol. 2021 Mar 18;11:646991. doi: 10.3389/fcimb.2021.646991 (PMC8044998; doi:10.3389/fcimb.2021.646991)

**Figure S1**

**A.**

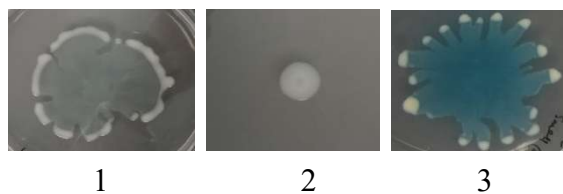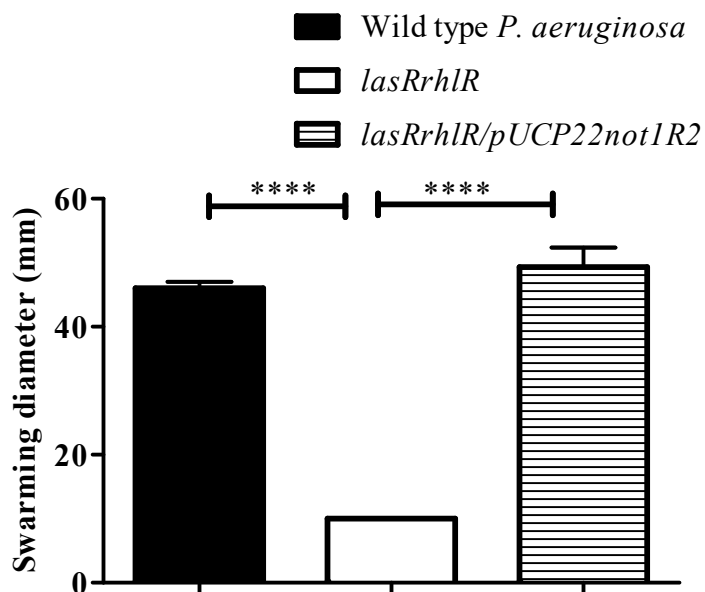

**B.**

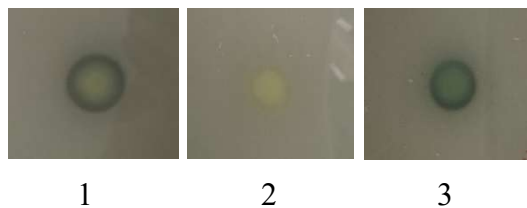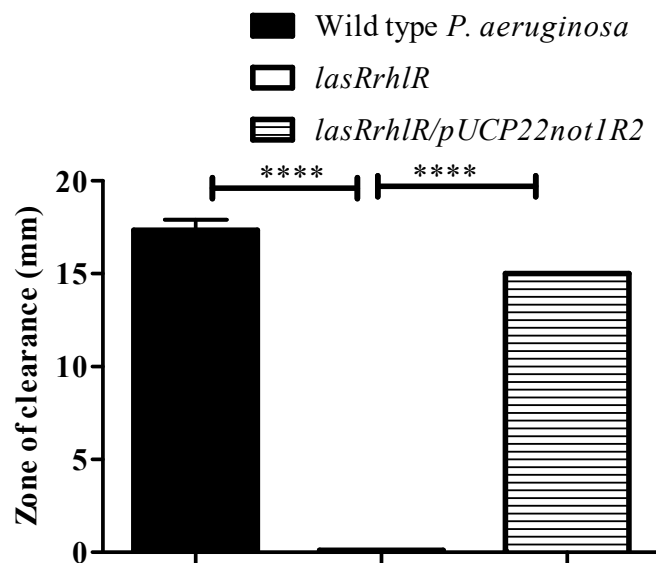

**C.**

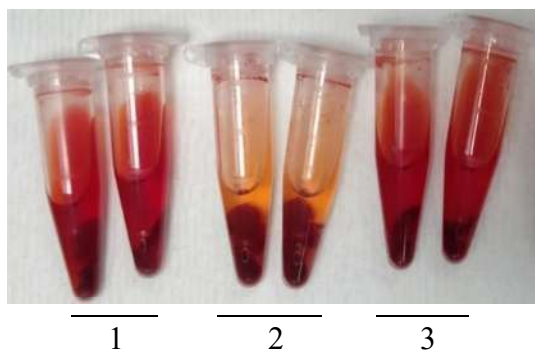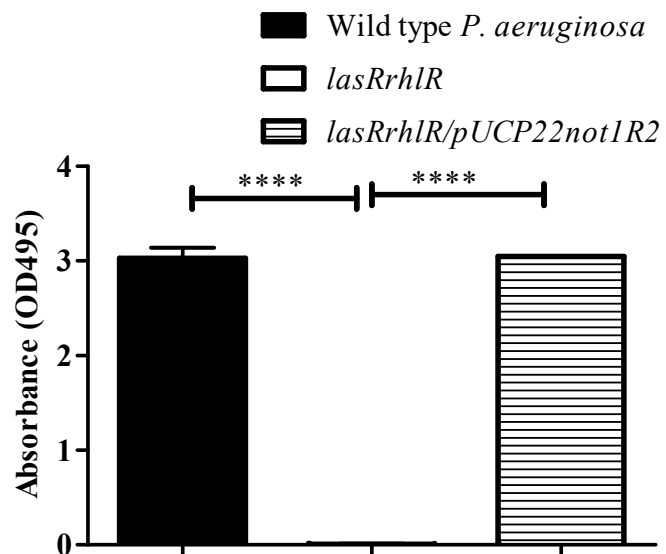

**Figure S2**

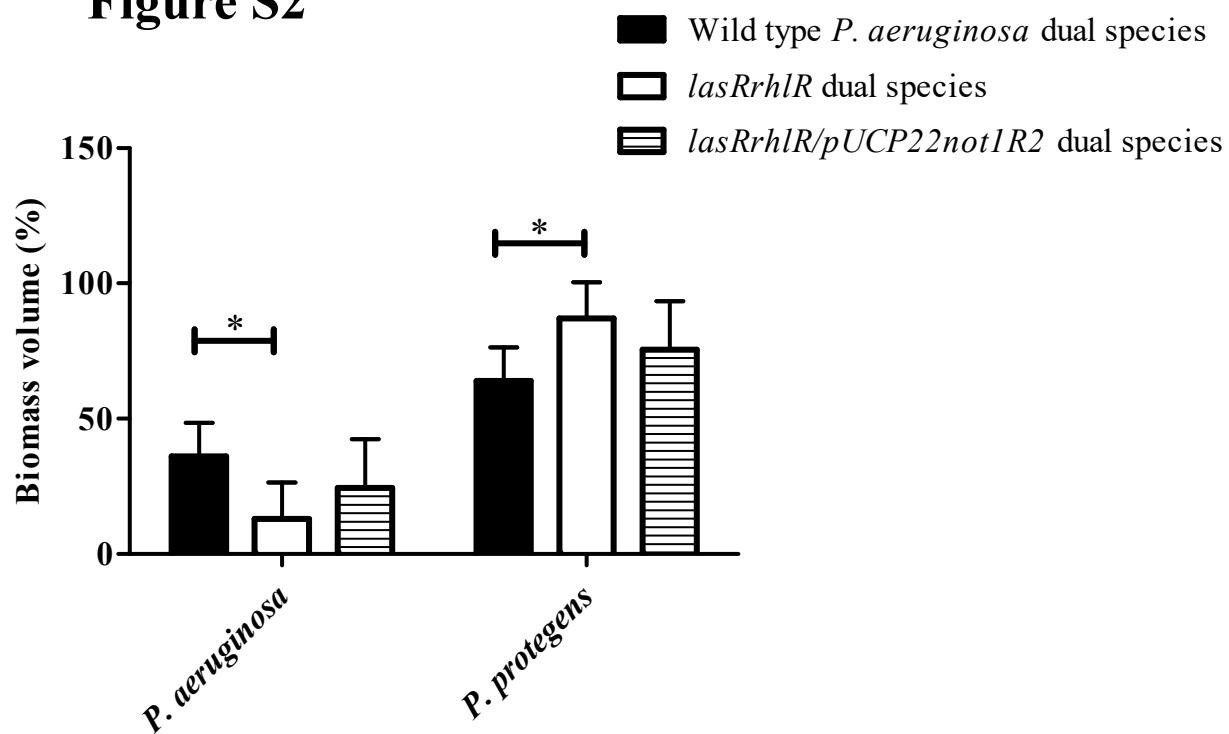

### Figure S3

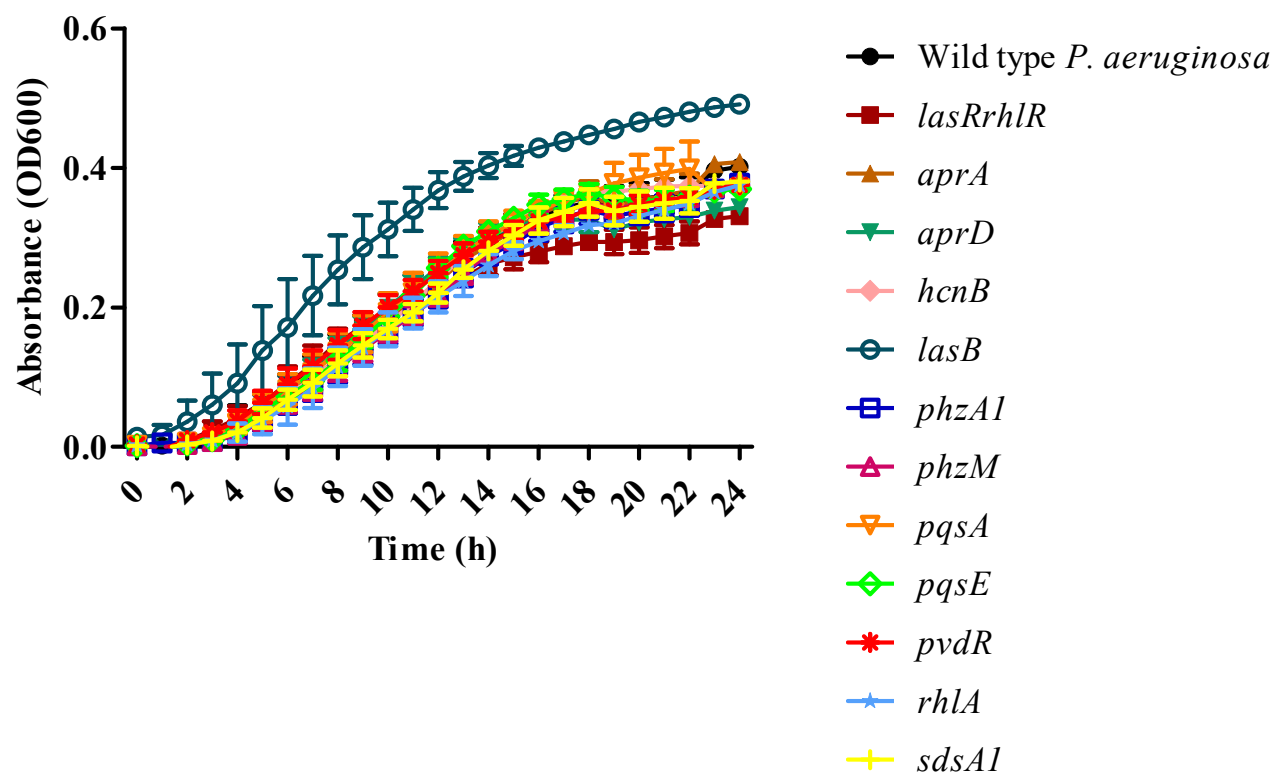

**Figure S4**

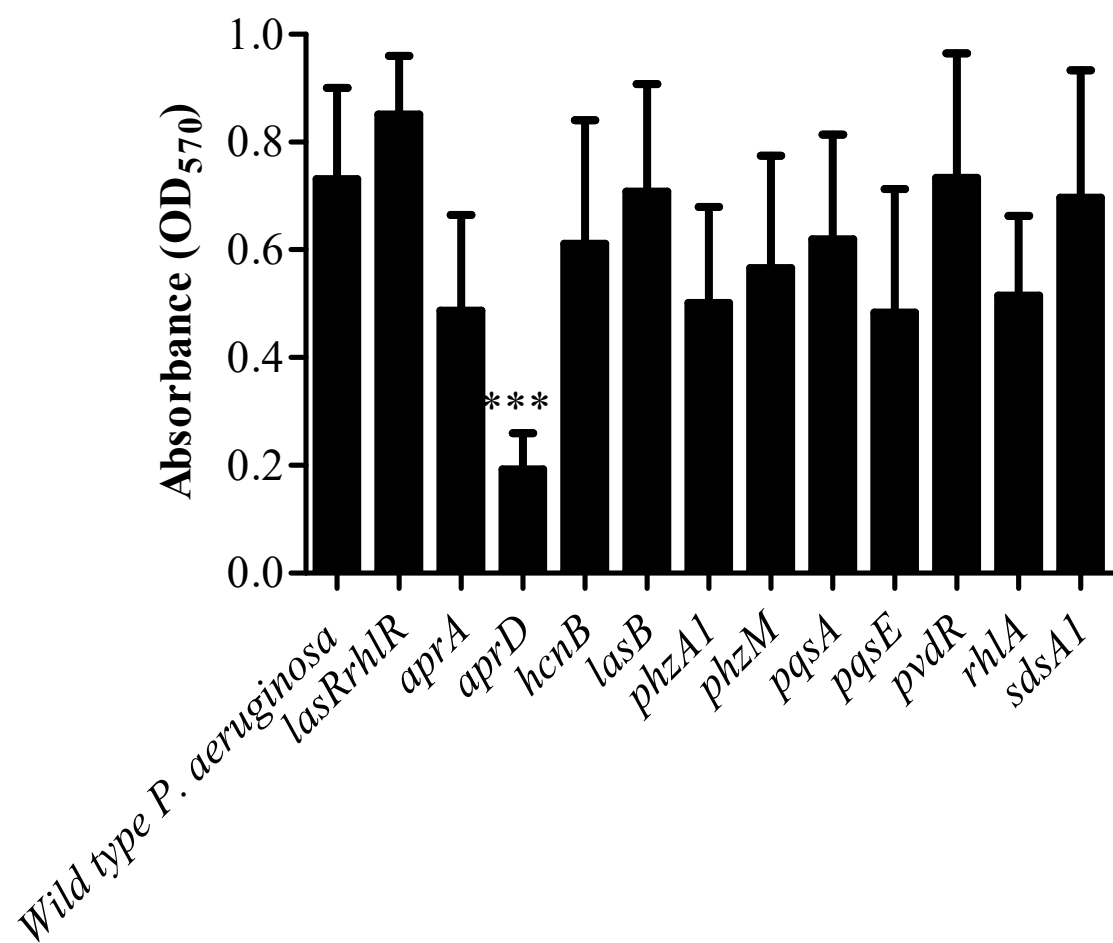

**Figure S5**

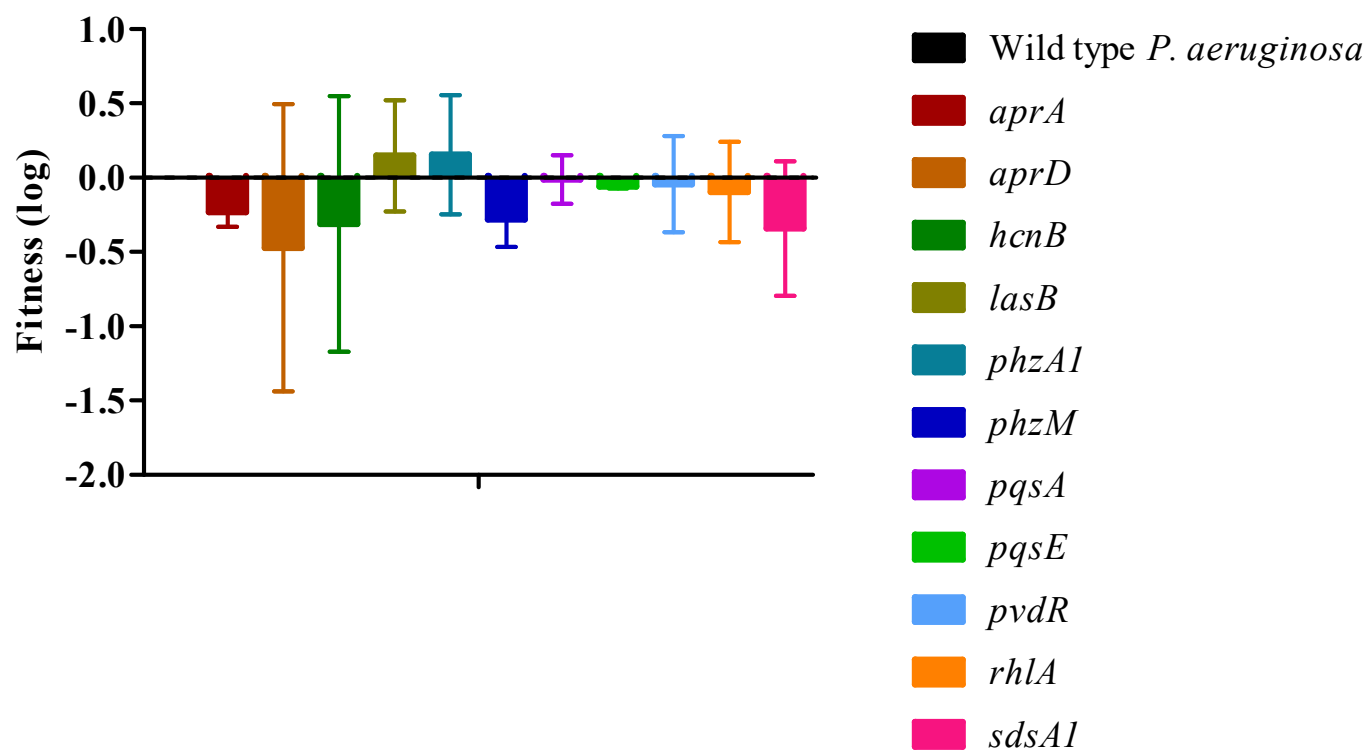

**Figure S6**

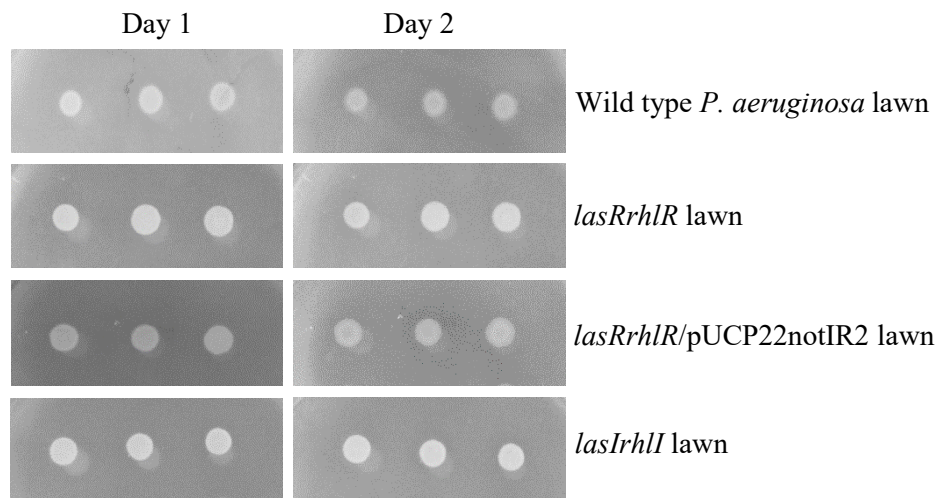

**Figure S7**

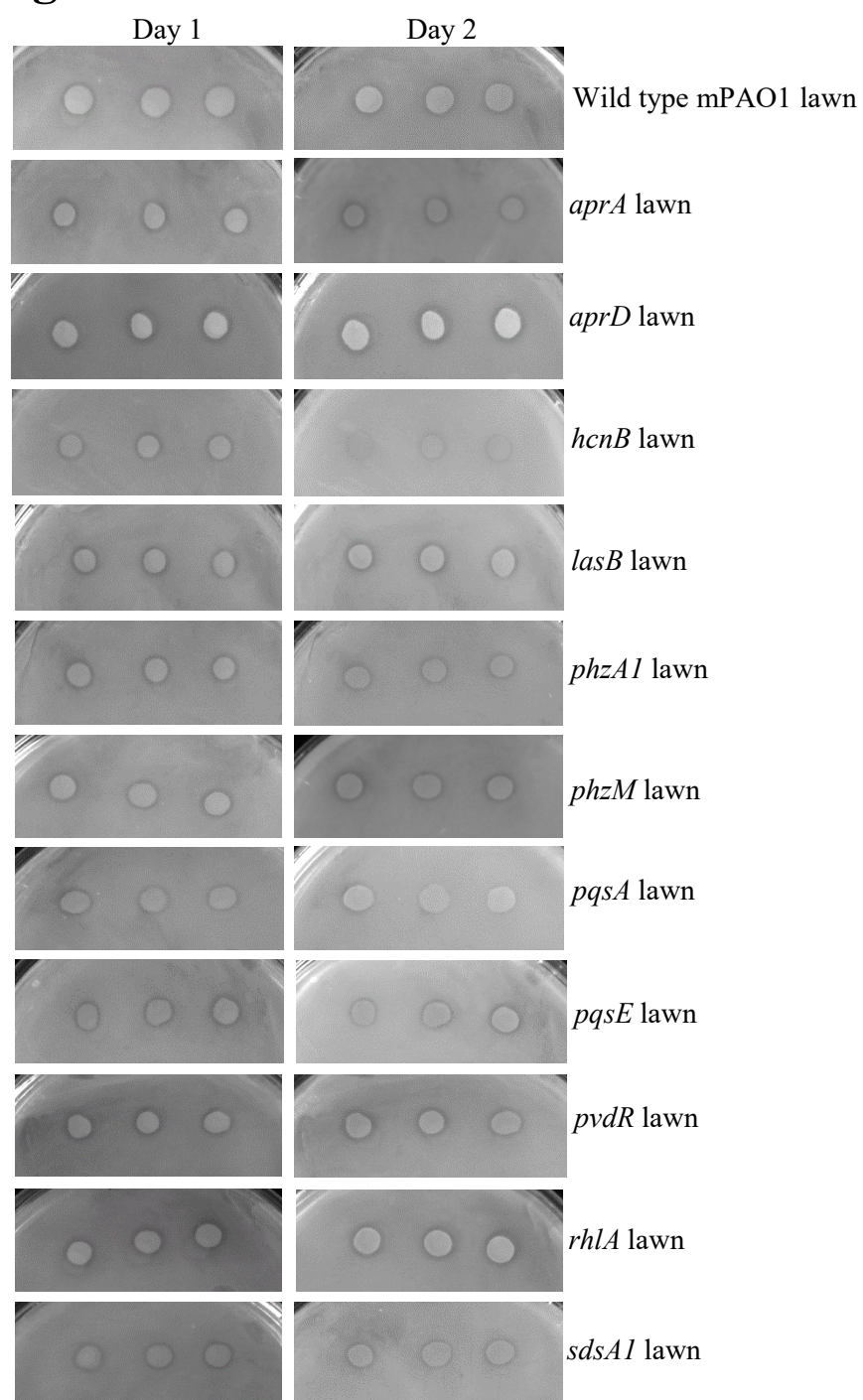

**Figure S8**

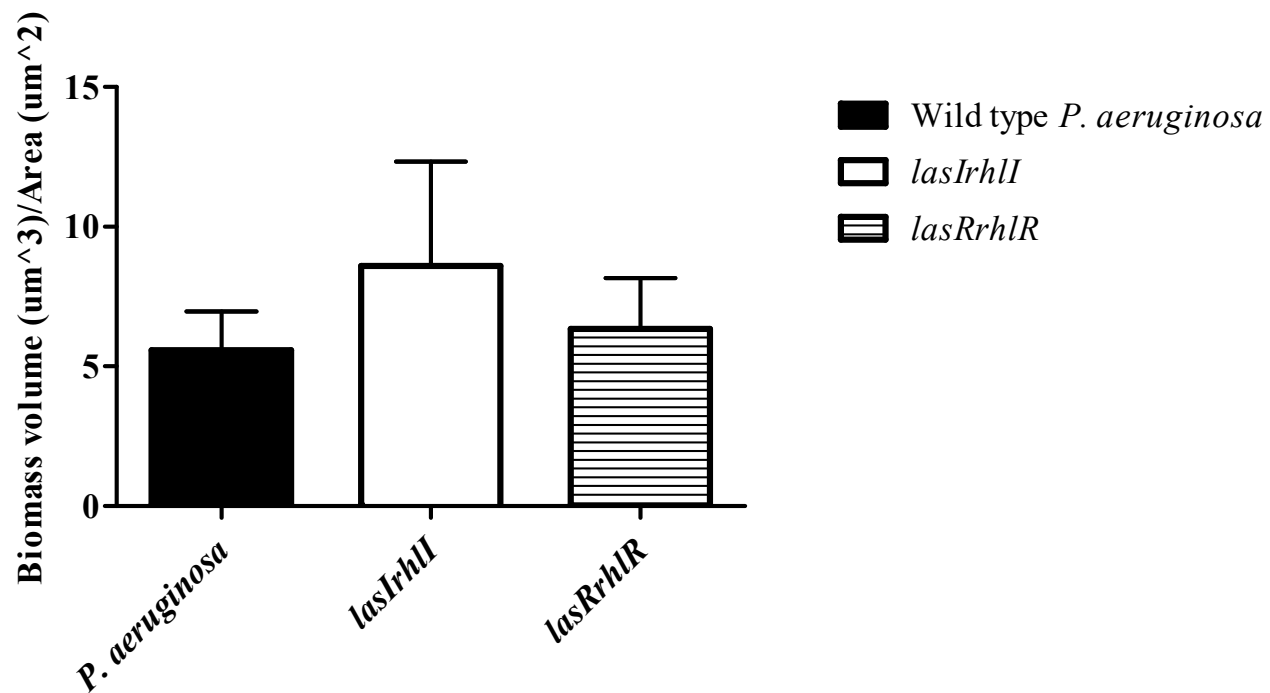

Supplement: Supplementary Figure 1 — QS regulated phenotypes of P. aeruginosa wild type, lasRrhlR mutant and the complemented strain. (A) Swarming motility of indicated strains on 0.5% swarm agar. Plates were incubated at 37°C for 16 h. Bars represent average diameter of the swarm area. (B) Protease activity of indicated strains on 1% skimmed milk containing LB agar plates. Plates were incubated at 37°C for 16 h. Bars represent average diameter of the zone of clearance around the colonies. (C) Elastase activity of indicated strains measured by mixing 100 µl of culture supernatant with 900 µl of Elastin-Congo Red containing buffer and incubating overnight at 37°C. Elastase activity was calculated by measuring the absorbance (OD495) of the supernatant. Bars represent OD495 values obtained after subtracting the values of medium control from the sample values. The data are averages from three independent biological replicates. **** denotes significant difference by One-way ANOVA followed by Tukey’s post-test (P<0.0001). 1, wild type P. aeruginosa; 2, lasRrhlR; 3, lasRrhlR/pUCP22notI+R2. [file DataSheet_1.pdf]
