# Supplementary material for: N-Acyl Homoserine Lactone-Mediated Quorum Sensing Regulates Species Interactions in Multispecies Biofilm Communities
Source: Front Cell Infect Microbiol. 2021 Mar 18;11:646991. doi: 10.3389/fcimb.2021.646991 (PMC8044998; doi:10.3389/fcimb.2021.646991)
Supplement: Supplementary file 2 [file Table_1.docx]

Table S1. Primers used in this study.

| Name | Primer sequence (5’-3’) | Used for |
| --- | --- | --- |
| LasRP1 | AGCTCGGTACCCGGGGCCGAACTGGAAAAGTGGCT | Amplification of upstream region of *lasR* |
| LasRP2 | CGCCGACCTGAGAGGCAAGAAGCGCTACGTTCTTCTTAAACTATTAACCA | Amplification of upstream region of *lasR* |
| LasRP3 | TTTAAGAAGAACGTAGCGCTTCTTGCCTCTCAGGTCGGCGAGCTG | Amplification of downstream region of *lasR* |
| LasRP4 | CGACGGCCAGTGCCAAGAACGCGCCGGCGCGAGCC | Amplification of downstream region of *lasR* |
| LasRFlankF | CCTATGCGCCGCCGTTGCAG | Confirmation sequencing |
| LasRFlankR | CGGCGCCCTGGTCCGGGGAT | Confirmation sequencing |
| RhlRP1 | AGCTCGGTACCCGGGGCAGCGCGCCTACGCGCCAC | Amplification of upstream region of *rhlR* |
| RhlRP2 | CACATGAGGGGGAAGACTAAAGGAGGATGAACGGCAGGCAACCTGCCAG | Amplification of upstream region of *rhlR* |
| RhlRP3 | CTGGCAGGTTGCCTGCCGTTCATCCTCCTTTAGTCTTCCCCCTCATGTG | Amplification of downstream region of *rhlR* |
| RhlRP4 | CGACGGCCAGTGCCAGCTGGCCAGCAGCGCAAG | Amplification of downstream region of *rhlR* |
| RhlRFlankF | ACGCCATGGCCCTGCGCG | Confirmation sequencing |
| RhlRFlankR | GGGTGCGGTAGCTGAAGGGCTTGT | Confirmation sequencing |
| RhlIP1 | AGCTCGGTACCCGGGTGGCGCGCGACCAGCAGAAC | Amplification of upstream region of *rhlI* |
| RhlIP2 | GCGCGAAACGGCTGACGACCGACCAAGTCCCCGTGTCGTGCC | Amplification of upstream region of *rhlI* |
| RhlIP3 | GGCACGACACGGGGACTTGGTCGGTCGTCAGCCGTTTCGCGC | Amplification of downstream region of *rhlI* |
| RhlIP4 | CGACGGCCAGTGCCACACCCTCGCCGCCCCTGTA | Amplification of downstream region of *rhlI* |
| RhlIFlankUP | GAGCCGGATGCTCTGGAACGAGGC | Confirmation sequencing |
| RhlIFlankDN | CGCATGGACCAGGCACCAGGATGG | Confirmation sequencing |
| LasIUPP1# | atgattacgaattcgagctcggtaccGGGAGATATCGGTTATCTGCAAC | Amplification of upstream region of *lasI* |
| LasIUPP2# | gcgcttttgaagctaattcgCTTCACTTCCTCCAAATAGGAAG | Amplification of upstream region of *lasI* |
| LasIDNP3# | acttcaagatccccaattcgCGGGGACCTGTCGGCTCGCGCCG | Amplification of downstream region of *lasI* |
| LasIDNP4# | acgacggccagtgccaagctTCCGCAGGAGGCGCGCCAGCTGAC | Amplification of downstream region of *lasI* |
| LasIFlankUP | GGTGCCGGACTGGCCTTCGA | Confirmation sequencing |
| LasIFlankDN | GGCCGCCAGTGTCCGCAATG | Confirmation sequencing |
| RhIRHinF2 | GGGGAAGCTTTCAGATGAGACCCAGCGCCGC | Complementation |
| RhlRR2 | CACTTTTCCAGTTCGGGCAGCGCGCCTACGCGCCA | Complementation |
| LasRF2 | GCGTAGGCGCGCTGCCCGAACTGGAAAAGTGGCTATGTCGCCGG | Complementation |
| LasREcoR2 | GGGGGAATTCTCAGAGAGTAATAAGACCCAAATTAACGGCCA | Complementation |
| pK18F | TGCTTCCGGCTCGTATGTTG | Verification of deletion construct |
| pK18R | GCGAAAGGGGGATGTGCTG | Verification of deletion construct |

#-lower and upper case nucleotides indicate the different parts of a hybrid primer homologous to two different regions
